# Supplementary material for: Prolonged Water-Only Fasting Followed by a Whole-Plant-Food Diet Is a Potential Long-Term Management Strategy for Hypertension and Obesity
Source: Nutrients. 2024 Nov 20;16(22):3959. doi: 10.3390/nu16223959 (PMC11597177; doi:10.3390/nu16223959)
Supplement: Supplementary file 1 [file nutrients-16-03959-s001.zip › nutrients-3298859-supplementary.pdf]

Supplementary Materials

1. Supplemental Methods

Method S1. Demographics, Medical Diagnoses, Medication Use, and Anthropometric Measurements

Demographic information (age, sex), current and past medical diagnoses, and current medication use was collected by interview at BL and self-reported at 6wFU and 12mFU visits using an online survey. Height (cm) was measured once at BL with a digital wall mounted stadiometer (DS5100, Doran Scales Inc., St. Charles, IL, USA) and used to calculate body mass index (BMI) at each time point. Participants were instructed to wear only one layer of clothing and remove shoes and any pocket items for BW measurements. BW (kg) was measured using a digital body scale (BWB 800A Class III, Tanita Corporation of American Inc., Arlington Heights, IL, USA) while onsite and with the provided digital floor scale (WW26, Conair LLC, Stamford, CT, USA) while offsite. AC (cm) was measured horizontally on a bare abdomen at the minimal waistline and parallel to the floor with a tension-sensitive, non-elastic tape (Gullick II, Model 67019, Country Technology Inc., Gay Mills, WI, USA) while onsite or with the provided retractable tape measure while offsite. The measurement was read in the midaxillary line and at the end of a normal expiration. BP was measured while the participant was resting in a seated position and their left arm elevated to heart level for at least five minutes. A digital blood pressure device (Welch Allyn-Connex ProBP 3400, Hill-Rom Holding Inc. Chicago, IL, USA) was used while onsite and a blood pressure monitor (BP3GX1, Microlife USA Inc, Clearwater, FL, USA) for the offsite visits. Participants received thorough education as well as written instructions in order to provide self-reported measurements at 6wFU and 12mFU visits.

Method S2. Biospecimen Collection

For onsite visits, serum and plasma blood samples as well as 24-hour urine were collected and sent to LabCorp (LabCorp, Burlington, NC, USA). For offsite visits, the samples were collected directly at a LabCorp local to the participant. All blood samples were drawn by a certified phlebotomist. Blood samples were drawn while onsite by a certified phlebotomist at BL, EOF, and EOR and at a local LabCorp for the off-site 6wFU and 12mFU visits. Blood collection took place in the morning, before the consumption of caloric food or liquid but with the instruction to consume 1-2 cups of water prior to arrival. While in a seated position, blood was collected into one vacutainer tube for sera (Red top, 16 × 100, 10 mL, silica, BD, Mississauga, ON, Canada) and one vacutainer tube with EDTA for whole blood (Lavender top, 13x75, 4.0 ml, K2EDTA, BD, Mississauga, ON, Canada). The lavender top vacutainer was placed into an ice/water bath immediately after collection and stored at 4◦C until it was sent to a commercial laboratory (LabCorp, Burlington, NC, USA) for complete blood count (CBC) analysis. LabCorp reports that CBC analysis was completed using an Auto cell counter with mixed technologies from Sysmex (Sysmex Asia Pacific Pte Ltd, Asia Green, Singapore). The red top vacutainer tube was incubated at room temperature for 30-50 min and centrifuged at 1500g for 10 minutes at 4°C (Model: Thermo Scientific Sorvall Legend X1R, Rotor: TX-400 Rotor Cross, Thermo Fisher Scientific Inc., Waltham, MA, USA). The serum was separated and stored at 4◦C until it was sent to LabCorp for comprehensive metabolic panel (CMP) analysis, lipid panel (total cholesterol, HDL-, LDL-, VLDL-cholesterol, triglycerides), C-reactive protein (CRP), gamma-glutamyl transferase (GGT), and insulin. LabCorp reports that CMP was measured using Ion Selective Electrode (ISE), colorimetric, enzymatic, and kinetic methods from Roche Diagnostics (Roche Diagnostics, Indianapolis, IN, USA), lipid panel by colorimetric and enzymatic methods, insulin by electrochemiluminescence immunoassays (ECLIA), CRP by high sensitivity immunochemiluminometric assays (ICMA), and GGT by kinetic methods from Roche Diagnostics (Roche Diagnostics, Indianapolis, IN, USA).

Participants collected 24-hour urine samples at BL, EOF, EOR, 6wFU, and 12mFU visits. The collection started after the first void was discarded and subsequent voids were collected into one or two additive-free urine containers (LabCorp) and stored at 4◦C for the next 24 hours. After completion, 24-hour urine samples were combined and/or inverted to ensure homogeneity and the total volume was assessed. 50 ml was transferred into a cup and a dipstick urinalysis (Siemens Multistix 10SG [Siemens Medical Solutions USA, Inc., Malvern, PA, USA] or Mission

Urinalysis Strips [ACON Laboratories, Inc., San Diego, CA, USA]) was performed at BL, EOF, and EOR to measure leukocytes, nitrite, urobilinogen, protein, pH, blood, specific gravity, ketone, bilirubin, and glucose. 50ml urine sample was sent to LabCorp for analysis of Sodium/24h Urine, Protein Total/24h Urine, Potassium/24 h Urine, Creatinine/24h Urine, and Albumin/24h Urine at BL, EOF, EOR. LabCorp reports urine was analyzed using Ion Selective Electrode (ISE), colorimetric, enzymatic, and kinetic methods from Roche Diagnostics. USG was measured in duplicate with 250 µL urine using a refractometer (Sper Scientific; Model 300036; Scottsdale, AZ).

BMI (kg/m2) was calculated using the formula[21]: weight (kg) ÷ height2 (m2); the homeostatic model assessment of insulin resistance (HOMA-IR) was calculated using the formula[22]: insulin (µU/L) × glucose (nmol/L)/22.5; and fatty liver index (FLI) was calculated using the formula[23]:

FLI= ((e^(0.953* 〖log〗_e TG + 0.139*BMI+0.718* 〖log〗_e GGT+0.053*AC-15.745)))/((1+ e^(0.953* 〖log〗_e TG + 0.139*BMI+0.718* 〖log〗_e GGT+0.053*AC-15.745)))*100

2. Supplemental Tables

Table S1. Baseline ICD-10 Diagnosis & Code by Sex

Diagnosis*	Code	Overall<br>N = 29	Female<br>N = 19	Male<br>N = 10
Mixed hyperlipidemia	**E78.2**	10 (34%)	7 (37%)	3 (30%)
Hypothyroidism, unspecified	**E03.9**	3 (10%)	3 (16%)	0 (0%)
Insomnia, unspecified	**G47.00**	3 (10%)	1 (5.3%)	2 (20%)
Gastro-esophageal reflux disease without esophagitis	**K21.9**	3 (10%)	3 (16%)	0 (0%)
Localized edema	**R60.0**	3 (10%)	2 (11%)	1 (10%)
Prediabetes	**R73.03**	3 (10%)	3 (10%)	0 (0%)
Leiomyoma of uterus, unspecified	**D25.9**	2 (6.9%)	2 (11%)	0 (0%)
Hyperlipidemia, unspecified	**E78.5**	2 (6.9%)	2 (11%)	0 (0%)
Major depressive disorder, recurrent, moderate	**F33.1**	2 (6.9%)	1 (5.3%)	1 (10%)
Adjustment disorder with anxiety	**F43.22**	2 (6.9%)	2 (11%)	0 (0%)
Obstructive sleep apnea	**G47.33**	2 (6.9%)	1 (5.3%)	1 (10%)
Asymptomatic varicose veins of bilateral lower extremities	**I83.93**	2 (6.9%)	1 (5.3%)	1 (10%)
Allergic rhinitis due to pollen	**J30.1**	2 (6.9%)	0 (0%)	2 (20%)
Pain in left hip	**M25.552**	2 (6.9%)	2 (11%)	0 (0%)
Other cervical disc degeneration, unspecified cervical region	**M50.30**	2 (6.9%)	1 (5.3%)	1 (10%)
Other intervertebral disc degeneration, lumbar region	**M51.36**	2 (6.9%)	1 (5.3%)	1 (10%)
Cervicalgia	**M54.2**	2 (6.9%)	2 (11%)	0 (0%)
Low back pain	**M54.5**	2 (6.9%)	2 (11%)	0 (0%)
Stress incontinence	**N39.3**	2 (6.9%)	2 (11%)	0 (0%)

Benign prostatic hyperplasia with lower urinary tract symptoms	**N40.1**	2 (6.9%)	0 (0%)	2 (20%)
Menopausal and female climacteric states	**N95.1**	2 (6.9%)	2 (11%)	0 (0%)
Rash and other nonspecific skin eruption	**R21**	2 (6.9%)	2 (11%)	0 (0%)
Nocturia	**R35.1**	2 (6.9%)	0 (0%)	2 (20%)
Basal cell carcinoma of skin, unspecified	**C44.91**	1 (3.4%)	0 (0%)	1 (10%)
Anemia, unspecified	**D64.9**	1 (3.4%)	1 (5.3%)	0 (0%)
Nontoxic multinodular goiter	**E04.2**	1 (3.4%)	1 (5.3%)	0 (0%)
Thyroiditis	**E06**	1 (3.4%)	1 (5.3%)	0 (0%)
Type 2 diabetes mellitus with hyperglycemia	**E11.65**	1 (3.4%)	1 (5.3%)	0 (0%)
Testicular hypofunction	**E29.1**	1 (3.4%)	0 (0%)	1 (10%)
Alcohol dependence with alcohol-induced sleep disorder	**F10.282**	1 (3.4%)	1 (5.3%)	0 (0%)
Post-traumatic stress disorder, chronic	**F43.12**	1 (3.4%)	1 (5.3%)	0 (0%)
Migraine, unspecified, not intractable, without status migrainosus	**G43.909**	1 (3.4%)	1 (5.3%)	0 (0%)
Episodic cluster headache, not intractable	**G44.019**	1 (3.4%)	1 (5.3%)	0 (0%)
Unspecified glaucoma	**H40.9**	1 (3.4%)	1 (5.3%)	0 (0%)
Left bundle-branch block, unspecified	**I44.7**	1 (3.4%)	1 (5.3%)	0 (0%)
Cardiac arrhythmia, unspecified	**I49.9**	1 (3.4%)	1 (5.3%)	0 (0%)
Varicose veins of unspecified lower extremity with inflammation	**I83.10**	1 (3.4%)	1 (5.3%)	0 (0%)
Allergic rhinitis, unspecified	**J30.9**	1 (3.4%)	1 (5.3%)	0 (0%)
Unspecified asthma, uncomplicated	**J45.909**	1 (3.4%)	1 (5.3%)	0 (0%)
Diaphragmatic hernia without obstruction or gangrene	**K44.9**	1 (3.4%)	1 (5.3%)	0 (0%)
Rectal prolapse	**K62.3**	1 (3.4%)	1 (5.3%)	0 (0%)
Polyp of colon	**K63.5**	1 (3.4%)	1 (5.3%)	0 (0%)
Calculus of gallbladder without cholecystitis without obstruction	**K80.20**	1 (3.4%)	0 (0%)	1 (10%)
Local infection of the skin and subcutaneous tissue, unspecified	**L08.9**	1 (3.4%)	1 (5.3%)	0 (0%)
Dermatitis, unspecified	**L30.9**	1 (3.4%)	1 (5.3%)	0 (0%)
Lichen sclerosus et atrophicus	**L90.0**	1 (3.4%)	1 (5.3%)	0 (0%)
Bilateral primary osteoarthritis of knee	**M17.0**	1 (3.4%)	1 (5.3%)	0 (0%)
Unspecified osteoarthritis, unspecified site	**M19.90**	1 (3.4%)	1 (5.3%)	0 (0%)
Pain in unspecified joint	**M25.50**	1 (3.4%)	1 (5.3%)	0 (0%)

Pain in right shoulder	**M25.511**	1 (3.4%)	0 (0%)	1 (10%)
Pain in right hip	**M25.551**	1 (3.4%)	1 (5.3%)	0 (0%)
Pain in unspecified hip	**M25.559**	1 (3.4%)	0 (0%)	1 (10%)
Pain in right knee	**M25.561**	1 (3.4%)	1 (5.3%)	0 (0%)
Pain in right hand	**M79.641**	1 (3.4%)	0 (0%)	1 (10%)
Pain in left hand	**M79.642**	1 (3.4%)	0 (0%)	1 (10%)
Age-related osteoporosis without current pathological fracture	**M81.0**	1 (3.4%)	1 (5.3%)	0 (0%)
Segmental and somatic dysfunction of cervical region	**M99.01**	1 (3.4%)	0 (0%)	1 (10%)
Segmental and somatic dysfunction of thoracic region	**M99.02**	1 (3.4%)	1 (5.3%)	0 (0%)
Segmental and somatic dysfunction of sacral region	**M99.04**	1 (3.4%)	1 (5.3%)	0 (0%)
Uterovaginal prolapse, unspecified	**N81.4**	1 (3.4%)	1 (5.3%)	0 (0%)
Palpitations	**R00.2**	1 (3.4%)	1 (5.3%)	0 (0%)
Cardiac murmur, unspecified	**R01.1**	1 (3.4%)	1 (5.3%)	0 (0%)
Abdominal distension (gaseous)	**R14.0**	1 (3.4%)	1 (5.3%)	0 (0%)
Inappropriate diet and eating habits	**Z72.4**	1 (3.4%)	1 (5.3%)	0 (0%)
Hormone replacement therapy	**Z79.890**	1 (3.4%)	1 (5.3%)	0 (0%)
Personal history of malignant neoplasm of brain	**Z85.841**	1 (3.4%)	1 (5.3%)	0 (0%)
Personal history of other diseases of the musculoskeletal system and connective tissue	**Z87.39**	1 (3.4%)	0 (0%)	1 (10%)
Personal history of urinary calculi	**Z87.442**	1 (3.4%)	0 (0%)	1 (10%)
Presence of aortocoronary bypass graft	**Z95.1**	1 (3.4%)	1 (5.3%)	0 (0%)
Presence of coronary angioplasty implant and graft	**Z95.5**	1 (3.4%)	1 (5.3%)	0 (0%)
Presence of right artificial knee joint	**Z96.651**	1 (3.4%)	0 (0%)	1 (10%)

Participants may have had more than one diagnoses. ICD, international classification of disease;[1] N, total number; %, percent. *Preexisting conditions, not including hypertension.

Table S2. SOS-Free Diet Screener Serving Quantity Reported by Category at BL, 6wkFU, and 12mFU

	**N (%)**											
**Number of Servings**	**0 per month**	**1 per month**	**2-3 per month**	**1 per week**	**2 per week**	**3-4 per week**	**5-6 per week**	**1 per day**	**2 per day**	**3 per day**	**4 per day**	**≥ 5 per day**
						**Vegetables**						
**BL**	0 (0%)	0 (0%)	0 (0%)	0 (0%)	0 (0%)	4 (14%)	0 (0%)	5 (17%)	4 (14%)	6 (21%)	4 (14%)	6 (21%)
**6wkFU**	0 (0%)	0 (0%)	0 (0%)	0 (0%)	0 (0%)	1 (4%)	0 (0%)	2 (8%)	4 (15%)	5 (19%)	6 (23%)	8 (31%)
**12mFU**	0 (0%)	0 (0%)	0 (0%)	0 (0%)	0 (0%)	1 (5.9%)	1 (5.9%)	0 (0%)	4 (24%)	2 (12%)	4 (24%)	5 (29%)

**Fruits**												
**BL**	0 (0%)	1 (3%)	2 (7%)	2 (7%)	0 (0%)	2 (7%)	0 (0%)	2 (7%)	9 (31%)	5 (17%)	4 (14%)	2 (7%)
**6wkFU**	0 (0%)	0 (0%)	0 (0%)	0 (0%)	2 (8%)	0 (0%)	0 (0%)	4 (15%)	4 (15%)	9 (35%)	5 (19%)	2 (8%)
**12mFU**	0 (0%)	0 (0%)	0 (0%)	0 (0%)	0 (0%)	1 (5.9%)	0 (0%)	2 (12%)	5 (29%)	4 (24%)	1 (5.9%)	4 (24%)
**Avocado**												
**BL**	2 (7%)	3 (10%)	5 (17%)	3 (10%)	2 (7%)	8 (28%)	2 (7%)	4 (14%)	0 (0%)	0 (0%)	0 (0%)	0 (0%)
**6wkFU**	4 (15%)	2 (8%)	2 (8%)	2 (8%)	4 (15%)	4 (15%)	2 (8%)	6 (23%)	0 (0%)	0 (0%)	0 (0%)	0 (0%)
**12mFU**	2 (12%)	4 (24%)	3 (18%)	0 (0%)	2 (12%)	4 (24%)	1 (5.9%)	1 (5.9%)	0 (0%)	0 (0%)	0 (0%)	0 (0%)
**Whole Grain**												
**BL**	1 (3%)	1 (3%)	3 (10%)	3 (10%)	1 (3%)	6 (21%)	3 (10%)	4 (14%)	5 (17%)	1 (3%)	0 (0%)	0 (0%)
**6wkFU**	0 (0%)	0 (0%)	0 (0%)	0 (0%)	2 (8%)	5 (19%)	3 (12%)	6 (23%)	6 (23%)	2 (8%)	2 (8%)	0 (0%)
**12mFU**	0 (0%)	0 (0%)	2 (12%)	0 (0%)	1 (5.9%)	3 (18%)	1 (5.9%)	3 (18%)	4 (24%)	0 (0%)	3 (18%)	0 (0%)
**Whole Grain Flour**												
**BL**	6 (21%)	1 (3%)	1 (3%)	3 (10%)	7 (24%)	7 (24%)	1 (3%)	2 (7%)	1 (3%)	0 (0%)	0 (0%)	0 (0%)
**6wkFU**	4 (15%)	2 (8%)	3 (12%)	2 (8%)	7 (27%)	4 (15%)	1 (4%)	3 (12%)	0 (0%)	0 (0%)	0 (0%)	0 (0%)
**12mFU**	1 (5.9%)	2 (12%)	3 (18%)	1 (5.9%)	3 (18%)	4 (24%)	1 (5.9%)	1 (5.9%)	1 (5.9%)	0 (0%)	0 (0%)	0 (0%)
**Refined Grain**												
**BL**	7 (24%)	0 (0%)	5 (17%)	0 (0%)	3 (10%)	6 (21%)	3 (10%)	0 (0%)	0 (0%)	5 (17%)	0 (0%)	0 (0%)
**6wkFU**	9 (35%)	4 (15%)	5 (19%)	2 (8%)	5 (19%)	0 (0%)	0 (0%)	0 (0%)	0 (0%)	1 (4%)	0 (0%)	0 (0%)
**12mFU**	3 (18%)	2 (12%)	1 (5.9%)	3 (18%)	3 (18%)	3 (18%)	0 (0%)	0 (0%)	2 (12%)	0 (0%)	0 (0%)	0 (0%)
**Legumes**												
**BL**	1 (3%)	0 (0%)	2 (7%)	1 (3%)	2 (7%)	8 (28%)	4 (14%)	2 (7%)	5 (17%)	2 (7%)	2 (7%)	0 (0%)
**6wkFU**	0 (0%)	0 (0%)	0 (0%)	0 (0%)	2 (8%)	6 (23%)	6 (23%)	6 (23%)	2 (8%)	3 (12%)	1 (4%)	0 (0%)
**12mFU**	0 (0%)	0 (0%)	0 (0%)	1 (5.9%)	2 (12%)	3 (18%)	2 (12%)	4 (24%)	3 (18%)	1 (5.9%)	0 (0%)	1 (5.9%)
**Nuts and Seeds**												
**BL**	1 (3%)	2 (7%)	3 (10%)	2 (7%)	0 (0%)	2 (7%)	3 (10%)	7 (24%)	6 (21%)	3 (10%)	0 (0%)	0 (0%)
**6wkFU**	2 (8%)	0 (0%)	0 (0%)	1 (4%)	1 (4%)	7 (27%)	2 (8%)	5 (19%)	8 (31%)	0 (0%)	0 (0%)	0 (0%)
**12mFU**	1 (5.9%)	1 (5.9%)	1 (5.9%)	0 (0%)	1 (5.9%)	2 (12%)	0 (0%)	2 (12%)	7 (41%)	2 (12%)		
**Red Meat**												
**BL**	15 (52%)	3 (10%)	3 (10%)	2 (7%)	3 (10%)	2 (7%)	1 (3%)	0 (0%)	0 (0%)	0 (0%)	0 (0%)	0 (0%)
**6wkFU**	21 (81%)	1 (4%)	0 (0%)	2 (8%)	1 (4%)	0 (0%)	0 (0%)	1 (4%)	0 (0%)	0 (0%)	0 (0%)	0 (0%)
**12mFU**	13 (76%)	0 (0%)	1 (5.9%)	2 (12%)	0 (0%)	1 (5.9%)	0 (0%)	0 (0%)	0 (0%)	0 (0%)	0 (0%)	0 (0%)
**Poultry**												
**BL**	17 (59%)	0 (0%)	1 (3%)	4 (14%)	5 (17%)	1 (3%)	1 (3%)	0 (0%)	0 (0%)	0 (0%)	0 (0%)	0 (0%)
**6wkFU**	22 (85%)	1 (4%)	1 (4%)	2 (8%)	0 (0%)	0 (0%)	0 (0%)	0 (0%)	0 (0%)	0 (0%)	0 (0%)	0 (0%)
**12mFU**	12 (71%)	1 (5.9%)	1 (5.9%)	0 (0%)	2 (12%)	1 (5.9%)	0 (0%)	0 (0%)	0 (0%)	0 (0%)	0 (0%)	0 (0%)
**Fish**												
**BL**	10 (34%)	7 (24%)	5 (17%)	2 (7%)	2 (7%)	3 (10%)	0 (0%)	0 (0%)	0 (0%)	0 (0%)	0 (0%)	0 (0%)
**6wkFU**	19 (73%)	3 (12%)	2 (8%)	1 (4%)	1 (4%)	0 (0%)	0 (0%)	0 (0%)	0 (0%)	0 (0%)	0 (0%)	0 (0%)
**12mFU**	12 (71%)	2 (12%)	2 (12%)	0 (0%)	1 (5.9%)	0 (0%)	0 (0%)	0 (0%)	0 (0%)	0 (0%)	0 (0%)	0 (0%)

**Dairy**												
**BL**	14 (48%)	0 (0%)	1 (3%)	1 (3%)	2 (7%)	2 (7%)	1 (3%)	3 (10%)	2 (7%)	3 (10%)	0 (0%)	0 (0%)
**6wkFU**	16 (62%)	4 (15%)	2 (8%)	1 (4%)	0 (0%)	2 (8%)	0 (0%)	1 (4%)	0 (0%)	0 (0%)	0 (0%)	0 (0%)
**12mFU**	12 (71%)	2 (12%)	1 (5.9%)	0 (0%)	0 (0%)	1 (5.9%)	1 (5.9%)	0 (0%)	0 (0%)	0 (0%)	0 (0%)	0 (0%)
**Eggs**												
**BL**	13 (45%)	2 (7%)	2 (7%)	2 (7%)	4 (14%)	3 (10%)	2 (7%)	1 (3%)	0 (0%)	0 (0%)	0 (0%)	0 (0%)
**6wkFU**	22 (85%)	0 (0%)	2 (8%)	1 (4%)	1 (4%)	0 (0%)	0 (0%)	0 (0%)	0 (0%)	0 (0%)	0 (0%)	0 (0%)
**12mFU**	12 (71%)	3 (18%)	0 (0%)	1 (5.9%)	0 (0%)	1 (5.9%)	0 (0%)	0 (0%)	0 (0%)	0 (0%)	0 (0%)	0 (0%)
**Prepared Food with Salt**												
**BL**	3 (10%)	1 (3%)	0 (0%)	5 (17%)	3 (10%)	6 (21%)	4 (14%)	1 (3%)	2 (7%)	1 (3%)	1 (3%)	2 (7%)
**6wkFU**	6 (23%)	3 (12%)	7 (27%)	2 (8%)	3 (12%)	3 (12%)	1 (4%)	0 (0%)	0 (0%)	1 (4%)	0 (0%)	0 (0%)
**12mFU**	2 (12%)	2 (12%)	2 (12%)	3 (18%)	4 (24%)	2 (12%)	0 (0%)	1 (5.9%)	1 (5.9%)	0 (0%)	0 (0%)	0 (0%)
**Added Salt**												
**BL**	8 (28%)	2 (7%)	2 (7%)	1 (3%)	3 (10%)	3 (10%)	2 (7%)	2 (7%)	0 (0%)	1 (3%)	1 (3%)	4 (14%)
**6wkFU**	16 (62%)	1 (4%)	3 (12%)	1 (4%)	1 (4%)	2 (8%)	1 (4%)	1 (4%)	0 (0%)	0 (0%)	0 (0%)	0 (0%)
**12mFU**	7 (41%)	1 (5.9%)	1 (5.9%)	3 (18%)	2 (12%)	0 (0%)	1 (5.9%)	2 (12%)	0 (0%)	0 (0%)	0 (0%)	0 (0%)
**Cooked with Salt**												
**BL**	7 (24%)	2 (7%)	1 (3%)	0 (0%)	4 (14%)	5 (17%)	2 (7%)	1 (3%)	3 (10%)	1 (3%)	0 (0%)	3 (10%)
**6wkFU**	12 (46%)	5 (19%)	2 (8%)	2 (8%)	0 (0%)	1 (4%)	1 (4%)	2 (8%)	0 (0%)	1 (4%)	0 (0%)	0 (0%)
**12mFU**	8 (47%)	1 (5.9%)	3 (18%)	0 (0%)	0 (0%)	2 (12%)	2 (12%)	1 (5.9%)	0 (0%)	0 (0%)	0 (0%)	0 (0%)
**Prepared Food with Oil**												
**BL**	3 (10%)	1 (3%)	5 (17%)	4 (14%)	0 (0%)	0 (0%)	10 (34%)	1 (3%)	4 (14%)	1 (3%)	0 (0%)	0 (0%)
**6wkFU**	9 (35%)	6 (23%)	6 (23%)	2 (8%)	0 (0%)	0 (0%)	1 (4%)	1 (4%)	1 (4%)	0 (0%)	0 (0%)	0 (0%)
**12mFU**	3 (18%)	4 (24%)	2 (12%)	6 (35%)	0 (0%)	0 (0%)	1 (5.9%)	1 (5.9%)	0 (0%)	0 (0%)	0 (0%)	0 (0%)
**Added Oil**												
**BL**	16 (55%)	1 (3%)	4 (14%)	0 (0%)	1 (3%)	1 (3%)	1 (3%)	3 (10%)	0 (0%)	1 (3%)	1 (3%)	0 (0%)
**6wkFU**	19 (73%)	3 (12%)	1 (4%)	2 (8%)	1 (4%)	0 (0%)	0 (0%)	0 (0%)	0 (0%)	0 (0%)	0 (0%)	0 (0%)
**12mFU**	14 (82%)	0 (0%)	0 (0%)	0 (0%)	1 (5.9%)	2 (12%)	0 (0%)	0 (0%)	0 (0%)	0 (0%)	0 (0%)	0 (0%)
**Cooked with Oil**												
**BL**	9 (31%)	2 (7%)	4 (14%)	1 (3%)	1 (3%)	2 (7%)	3 (10%)	3 (10%)	0 (0%)	4 (14%)	0 (0%)	0 (0%)
**6wkFU**	17 (65%)	1 (4%)	5 (19%)	0 (0%)	1 (4%)	1 (4%)	0 (0%)	0 (0%)	1 (4%)	0 (0%)	0 (0%)	0 (0%)
**12mFU**	9 (53%)	1 (5.9%)	0 (0%)	3 (18%)	1 (5.9%)	3 (18%)	0 (0%)	0 (0%)	0 (0%)	0 (0%)	0 (0%)	0 (0%)
**Prepared Food with Added Sugar**												
**BL**	6 (21%)	1 (3%)	3 (10%)	1 (3%)	2 (7%)	6 (21%)	2 (7%)	3 (10%)	3 (10%)	1 (3%)	0 (0%)	1 (3%)
**6wkFU**	12 (50%)	3 (12%)	4 (17%)	1 (4.2%)	0 (0%)	2 (8%)	0 (0%)	2 (8%)	0 (0%)	0 (0%)	0 (0%)	0 (0%)
**12mFU**	4 (25%)	2 (12%)	3 (19%)	2 (12%)	1 (6.2%)	1 (6.2%)	0 (0%)	0 (0%)	2 (12%)	1 (6.2%)	0 (0%)	0 (0%)
**Added Sugar**												
**BL**	18 (62%)	1 (3%)	2 (7%)	1 (3%)	1 (3%)	0 (0%)	2 (7%)	3 (10%)	0 (0%)	0 (0%)	0 (0%)	1 (3%)
**6wkFU**	24 (92%)	0 (0%)	2 (8%)	0 (0%)	0 (0%)	0 (0%)	0 (0%)	0 (0%)	0 (0%)	0 (0%)	0 (0%)	0 (0%)
**12mFU**	13 (76%)	1 (5.9%)	1 (5.9%)	0 (0%)	0 (0%)	0 (0%)	1 (5.9%)	1 (5.9%)	0 (0%)	0 (0%)	0 (0%)	0 (0%)

						**Cooked with Added Sugar**						
**BL**	11 (38%)	1 (3%)	6 (21%)	3 (10%)	2 (7%)	1 (3%)	2 (7%)	2 (7%)	0 (0%)	1 (3%)	0 (0%)	0 (0%)
**6wkFU**	21 (81%)	2 (8%)	2 (8%)	0 (0%)	0 (0%)	1 (4%)	0 (0%)	0 (0%)	0 (0%)	0 (0%)	0 (0%)	0 (0%)
**12mFU**	9 (53%)	1 (5.9%)	1 (5.9%)	0 (0%)	0 (0%)	5 (29%)	0 (0%)	1 (5.9%)	0 (0%)	0 (0%)	0 (0%)	0 (0%)
						**B12**						
**BL**	6 (21%)	1 (3%)	0 (0%)	2 (7%)	1 (3%)	7 (24%)	0 (0%)	12 (41%)	0 (0%)	0 (0%)	0 (0%)	0 (0%)
**6wkFU**	3 (12%)	0 (0%)	3 (12%)	0 (0%)	2 (8%)	5 (19%)	2 (8%)	11 (42%)	0 (0%)	0 (0%)	0 (0%)	0 (0%)
**12mFU**	3 (18%)	0 (0%)	0 (0%)	3 (18%)	0 (0%)	4 (24%)	0 (0%)	7 (41%)	0 (0%)	0 (0%)	0 (0%)	0 (0%)

			**Additional Supplements**			
	**None**	**Omega-3**	**Vitamin D**	**Iodine**	**Probiotics**	**Other**
**BL**	5 (17%)	6 (21%)	23 (79%)	0 (0%)	4 (14%)	9 (31%)
**6wkFU**	7 (27%)	4 (15%)	18 (69%)	2 (7.4%)	3 (12%)	6 (23%)
**12mFU**	4 (24%)	7 (41%)	12 (71%)	2 (12%)	5 (29%)	5 (29%)

						**Alcohol**						
**BL**	11 (38%)	4 (14%)	3 (10%)	3 (10%)	4 (14%)	2 (7%)	1 (3%)	0 (0%)	0 (0%)	1 (3%)	0 (0%)	0 (0%)
**6wkFU**	18 (69%)	5 (19%)	1 (4%)	0 (0%)	0 (0%)	0 (0%)	1 (4%)	0 (0%)	0 (0%)	1 (4%)	0 (0%)	0 (0%)
**12mFU**	12 (71%)	3 (18%)	1 (5.9%)	1 (5.9%)	0 (0%)	0 (0%)	0 (0%)	0 (0%)	0 (0%)	0 (0%)	0 (0%)	0 (0%)
						**Caffeine**						
**BL**	8 (28%)	1 (3%)	1 (3%)	1 (3%)	3 (10%)	1 (3%)	0 (0%)	11 (38%)	3 (10%)	0 (0%)	0 (0%)	0 (0%)
**6wkFU**	17 (65%)	3 (12%)	0 (0%)	1 (4%)	2 (8%)	2 (8%)	0 (0%)	1 (4%)	0 (0%)	0 (0%)	0 (0%)	0 (0%)
**12mFU**	6 (35%)	0 (0%)	4 (24%)	1 (5.9%)	1 (5.9%)	2 (12%)	2 (12%)	1 (5.9%)	0 (0%)	0 (0%)	0 (0%)	0 (0%)
						**Tobacco**						
**BL**	29 (100%)	0 (0%)	0 (0%)	0 (0%)	0 (0%)	0 (0%)	0 (0%)	0 (0%)	0 (0%)	0 (0%)	0 (0%)	0 (0%)
**6wkFU**	26 (100%)	0 (0%)	0 (0%)	0 (0%)	0 (0%)	0 (0%)	0 (0%)	0 (0%)	0 (0%)	0 (0%)	0 (0%)	0 (0%)
**12mFU**	17	0 (0%)	0 (0%)	0 (0%)	0 (0%)	0 (0%)	0 (0%)	0 (0%)	0 (0%)	0 (0%)	0 (0%)	0 (0%)

Screener reports on previous 30 days. N (%); number (percentage) of participants who selected a given serving size at BL (baseline; N=29), 6wkFU (six-week follow-up; N=26), and 12mFU (12-month follow-up; N=17) visits. The mean (SD) total score was 12 (10), 6 (3), and 6 (4) at BL, 6wkFU, and 12mFU, respectively with scores ranging from zero (100% adherence) to 82 (0% adherence).

Table S3. Adverse Events Occurring in ≥ 10% of Participants by Treatment Stage and Grade

			Prefeeding			Fasting			Refeeding		
**AE Classification**	**N (%)**	**Total AEs**	**G1**	**G2**	**G3**	**G1**	**G2**	**G3**	**G1**	**G2**	**G3**
Fatigue	26 (90%)	79	2	0	0	46	14	0	12	5	0
Blood bicarbonate decreased†	21 (72%)	22	0	0	0	22	0	0	0	0	0
BUN/creatinine decreased*	18 (62%)	18	1	0	0	10	0	0	7	0	0
Hypertension	16 (55%)	27	0	1	0	4	14	7	0	1	0
Nausea	14 (48%)	15	0	0	0	11	2	0	2	0	0
Chloride Decreased*†	13 (45%)	13	0	0	0	13	0	0	0	0	0
Hypoglycemia†	11 (38%)	12	0	0	0	8	4	0	0	0	0
Creatinine increased	11 (38%)	11	0	0	0	7	0	0	4	0	0
Hypokalemia†	11 (38%)	11	0	0	0	11	0	0	0	0	0
Blood bilirubin increased†	10 (34%)	10	0	0	0	9	1	0	0	0	0

Dizziness	10 (34%)	24	0	0	0	22	1	0	1	0	0
Headache	9 (31%)	10	1	0	0	7	1	0	1	0	0
Insomnia	8 (28%)	8	0	0	0	6	1	0	1	0	0
BUN Decreased*	8 (28%)	8	0	0	0	3	0	0	5	0	0
Presyncope†	7 (24%)	10	0	0	0	0	10	0	0	0	0
AST increased	6 (21%)	7	0	0	0	5	0	0	2	0	0
Palpitations	6 (21%)	7	0	0	0	6	0	0	1	0	0
ALT increased	6 (21%)	6	0	0	0	5	0	0	1	0	0
GERD†	6 (21%)	6	0	0	0	5	1	0	0	0	0
Hypercalcemia	6 (21%)	6	0	0	0	5	0	0	1	0	0
eGFR decreased*	6 (21%)	6	0	0	0	4	0	0	2	0	0
RBC Increased*†	6 (21%)	6	0	0	0	6	0	0	0	0	0
Back pain	5 (17%)	6	0	1	0	3	1	0	1	0	0
Hemoglobin increased†	5 (17%)	5	0	0	0	5	0	0	0	0	0
Hyperglycemia	5 (17%)	5	0	0	0	1	0	0	4	0	0
Hyponatremia	4 (14%)	5	0	0	0	4	0	0	1	0	0
Albumin increased*	4 (14%)	5	0	0	0	2	0	0	3	0	0
Hematuria	4 (14%)	4	0	0	0	3	0	0	1	0	0
Myalgia	4 (14%)	4	0	0	0	2	0	0	2	0	0
White blood cell decreased	4 (14%)	4	0	0	0	1	0	0	2	1	0
Allergic rhinitis	4 (14%)	4	1	0	0	3	0	0	0	0	0
Diarrhea	3 (10%)	4	0	0	0	0	0	0	4	0	0
Neutrophil count decreased	3 (10%)	4	1	0	0	0	0	0	2	0	1
Abdominal pain†	3 (10%)	4	0	0	0	3	1	0	0	0	0
Arthralgia†	3 (10%)	3	0	0	0	2	1	0	0	0	0
Dry mouth†	3 (10%)	3	0	0	0	3	0	0	0	0	0
Rash maculopapular	3 (10%)	3	0	0	0	1	1	0	1	0	0
Ventricular arrhythmia†	3 (10%)	3	0	0	0	3	0	0	0	0	0
Hematocrit Increased*†	3 (10%)	3	0	0	0	3	0	0	0	0	0
A/G Ratio Increased*	3 (10%)	3	0	0	0	2	0	0	1	0	0
Flatulence†	3 (10%)	3	0	0	0	3	0	0	0	0	0

AE, adverse event; N (%), number (percent) of total participants (N=29) experiencing AE; Grade 1 (G1), mild; Grade 2 (G2), moderate; Grade 3 (G3), severe; Grade 4 (G4), life-threatening; Grade 5 (G5), death; BUN , blood urea nitrogen; AST, aspartate aminotransferase; ALT, alanine aminotransferase; GERD, gastroesophageal reflux disease; eGFR, estimated glomerular filtration rate; RBC, red blood cell; A/G, albumin/globulin. There were no G4 or G5 events. *AE terms are listed in the CTCAE as "other" and were specified and graded according to CTCAE guidelines [2]. †Fifteen AEs occurred only during fasting.

Table S4. Total Adverse Events by Classification, Grade, and Outcome

				Grade			Outcome		
**SOC**	**AE Term**	**N**	**Total**	**1**	**2**	**3**	**Recovered**	**Persisted**	**Unknown**
General disorders and administration site conditions	Fatigue	26	79	60	19		77	2	
Vascular disorders	Hypertension	16	27	4	16	7	27		
Nervous system disorders	Dizziness	10	24	23	1		23	1	
Investigations	Blood bicarbonate decreased	21	22	22			22		

Metabolism and nutrition disorders	BUN/creatinine decreased*	18	18	18			12	3	3
Gastrointestinal disorders	Nausea	14	15	13	2		14	1	
Metabolism and nutrition disorders	Chloride Decreased*	13	13	13			12		1
Metabolism and nutrition disorders	Hypoglycemia	11	12	8	4		12		
Investigations	Creatinine increased	11	11	11			9	1	1
Metabolism and nutrition disorders	Hypokalemia	11	11	11			11		
Investigations	Blood bilirubin increased	10	10	9	1		10		
Nervous system disorders	Headache	9	10	9	1		9	1	
Nervous system disorders	Presyncope	7	10		10		10		
Psychiatric disorders	Insomnia	8	8	7	1		7	1	
Metabolism and nutrition disorders	BUN Decreased*	8	8	8			5	2	1
Investigations	Aspartate aminotransferase increased	6	7	7			7		
Cardiac disorders	Palpitations	6	7	7			7		
Investigations	Alanine aminotransferase increased	6	6	6			4	2	
Gastrointestinal disorders	Gastroesophageal reflux disease	6	6	5	1		5	1	
Metabolism and nutrition disorders	Hypercalcemia	6	6	6			4	2	
Investigations	eGFR decreased*	6	6	6			5	1	
Investigations	RBC Increased*	6	6	6			6		
Musculoskeletal and connective tissue disorders	Back pain	5	6	4	2		6		
Investigations	Hemoglobin increased	5	5	5			5		
Metabolism and nutrition disorders	Hyperglycemia	5	5	5			4		1
Metabolism and nutrition disorders	Hyponatremia	4	5	5			5		
Metabolism and nutrition disorders	Albumin increased*	4	5	5			4		1

Renal and urinary disorders	Hematuria	4	4	4			4		
Musculoskeletal and connective tissue disorders	Myalgia	4	4	4			4		
Investigations	White blood cell decreased	4	4	3	1		3		1
Respiratory, thoracic and mediastinal disorders	Allergic rhinitis	4	4	4			1	3	
Gastrointestinal disorders	Diarrhea	3	4	4			2	1	1
Investigations	Neutrophil count decreased	3	4	3		1	2		2
Gastrointestinal disorders	Abdominal pain	3	4	3	1		4		
Musculoskeletal and connective tissue disorders	Arthralgia	3	3	2	1		1	2	
Gastrointestinal disorders	Dry mouth	3	3	3			3		
Skin and subcutaneous tissue disorders	Rash maculopapular	3	3	2	1		3		
Cardiac disorders	Ventricular arrhythmia	3	3	3			2	1	
Investigations	Hematocrit Increased*	3	3	3			3		
Metabolism and nutrition disorders	A/G Ratio Increased*	3	3	3			3		
Gastrointestinal disorders	Flatulence	3	3	3			3		
Nervous system disorders	Extrapyramidal disorder	2	2	2			2		
Metabolism and nutrition disorders	Hyperkalemia	2	2	2			1	1	
Metabolism and nutrition disorders	Hypophosphatemia	2	2	2					2
Musculoskeletal and connective tissue disorders	Neck pain	2	2	2			1	1	
Gastrointestinal disorders	Toothache	2	2	2				2	
Renal and urinary disorders	Urinary tract pain	2	2	2			2		
Infections and infestations	Vaginal infection	2	2	2			2		
Gastrointestinal disorders	Vomiting	2	2	2			2		
Investigations	MCHC increased*	2	2	2			2		

Investigations	Electrocardiogram QT corrected interval prolonged	2	2	2				1	1
Respiratory, thoracic and mediastinal disorders	Nasal congestion	1	2	2			1		1
Investigations	Alkaline phosphatase increased	1	1	1			1		
Musculoskeletal and connective tissue disorders	Buttock pain	1	1	1			1		
Cardiac disorders	Chest pain-cardiac	1	1	1			1		
Nervous system disorders	Concentration impairment	1	1	1			1		
Respiratory, thoracic and mediastinal disorders	Cough	1	1	1			1		
Eye disorders	Dry eye	1	1	1			1		
Skin and subcutaneous tissue disorders	Dry skin	1	1	1			1		
Gastrointestinal disorders	Dyspepsia	1	1	1			1		
Renal and urinary disorders	Dysuria	1	1	1			1		
General disorders and administration site conditions	Edema face	1	1	1			1		
Eye disorders	Hordeolum*	1	1	1			1		
Gastrointestinal disorders	Gastritis	1	1	1			1		
Metabolism and nutrition disorders	Hypermagnesemia	1	1	1					1
Metabolism and nutrition disorders	Hyperuricemia	1	1	1					1
Musculoskeletal and connective tissue disorders	Muscle weakness lower limb	1	1	1			1		
Infections and infestations	Papulopustular rash	1	1	1			1		
Investigations	Platelet count decreased	1	1	1			1		
Renal and urinary disorders	Proteinuria	1	1	1			1		
Respiratory, thoracic and mediastinal disorders	Sore throat	1	1	1			1		

Cardiac disorders	Tachycardia	1	1	1			1		
Renal and urinary disorders	Urinary frequency	1	1	1				1	
Infections and infestations	Urinary tract infection	1	1	1				1	
Renal and urinary disorders	Urine discoloration	1	1	1			1		
Reproductive system and breast disorders	Vaginal inflammation	1	1	1			1		
Ear and labyrinth disorders	Vertigo	1	1		1		1		
Eye disorders	Vision decreased	1	1		1		1		
Investigations	Lymphs Absolute Increased*	1	1	1			1		
Investigations	RDW Decreased*	1	1	1			1		
Investigations	RDW Increased*	1	1	1			1		
Metabolism and nutrition disorders	Anion Gap Increased*	1	1	1					1
Metabolism and nutrition disorders	Creatinine decreased*	1	1	1				1	
Metabolism and nutrition disorders	Insulin decreased*	1	1	1			1		
Renal and urinary disorders	Urine Analysis Positive leukocytes*	1	1	1					1
Renal and urinary disorders	Urinary hesitancy*	1	1	1			1		
	**Total**	**359**	**453**	**381**	**64**	**8**	**400**	**33**	**20**

N, total number of participants experiencing AE; SOC, System Organ Class; AE, adverse event; Grade 1, mild; Grade 2, moderate; Grade 3, severe; Grade 4, life-threatening; Grade 5, death. BUN, blood urea nitrogen; eGFR, estimated glomerular filtration rate; RBC, red blood cell; A/G, albumin/globulin; MCHC, mean corpuscular hemoglobin concentration; RDW, red blood cell distribution width. AEs described as persisted were all G1 events that had not resolved at EOR or 6wkFU. Of the 20 AEs with unknown outcome, 19 were G1 and 1 was G3. There were no Grade 4 or 5 events. *AE terms are listed in the CTCAE as "other" and were specified and graded according to CTCAE guidelines [2].

Table S5. CBC by Visit

	Median (IQR)				
	BL	EOF	EOR	FU	12mFU
**Hematocrit, %**					
All	42.4 (39.2, 45.3)	45.0 (42.9, 47.2)	43.1 (40.3,45.6)	41.4 (40.2, 44.1)	43.3 (38.9, 46.0)
34.0-46.6 (female)	40.7 (38.2, 42.5)	43.4 (42.0, 45.0)	42.2 (38.5, 43.5)	40.4 (39.0, 41.0)	40.5 (38.9, 43.4)
37.5-51.0 (male)	46.8 (44.6, 49.7)	48.7 (46.5, 51.4)	46.4 (44.6, 48.4)	44.3 (42.8, 48.1)	47.7 (46.8, 49.0)
**Hemoglobin, g/dL**					
All	14.4 (12.8, 15.0)	15.6 (14.6, 16.6)	15.0 (13.5, 15.6)	13.9 (13.1, 15.0)	14.1 (13.0, 15.3)
11.1-15.9 (female)	14.2 (12.5, 14.4)	14.9 (14.1, 15.6)	14.3 (13.2, 15.4)	13.4 (12.6, 13.9)	13.4 (12.9, 14.2)
13.0-17.7 (male)	16.0 (14.6, 16.9)	17.0 (16.4, 17.8)	15.6 (15.1, 16.6)	15.3 (14.2, 16.4)	16.4 (15.8, 16.6)
**MCV, fL**					
79-97	89 (87, 92)	88 (85, 90)	89 (86, 91)	92 (89, 95)	92 (90, 95)

**MCH, pg**					
26.6-33.0	30.0 (29.2, 31.3)	30.9 (29.3, 31.5)	30.6 (29.7, 31.4)	30.8 (29.6, 31.4)	30.7 (29.6, 31.3)
**MCHC, g/dL**					
31.5-35.7	33.9 (33.1, 34.4)	34.5 (34.0, 35.1)	34.3 (33.5, 34.6)	33.3 (32.7, 34.0)	33.3 (32.6, 33.7)
**RDW, %**					
All	12.6 (12.1, 13.3)	12.8 (12.1, 13.4)	12.8 (12.3, 13.5)	13.1 (12.6, 13.8)	12.7 (12.3, 13.3)
11.7-15.4 (female)	12.6 (12.2, 13.1)	12.8 (12.3, 13.6)	12.8 (12.6, 13.5)	13.2 (12.7, 13.8)	12.7 (12.3, 13.2))
11.6-15.4 (male)	12.4 (11.9, 13.2)	12.4 (12.0, 13.2)	12.6 (11.9, 13.3)	13.0 (12.6, 13.4)	13.2 (12.6, 13.3)
**Platelets, x10^3/µL**					
150-450	254 (223, 285)	240 (204, 304)	222 (204, 292)	235 (219, 285)	266 (220, 307)
**RBC count, x10^6/µL**					
All	4.74 (4.43, 5.07)	5.04 (4.87, 5.39)	4.89 (4.50, 5.13)	4.55 (4.26, 4.87)	4.62 (4.24, 4.99)
3.77-5.28 (female)	4.48 (4.27, 4.78)	4.92 (4.74, 5.04)	4.77 (4.30, 5.00)	4.44 (4.12, 4.66)	4.41 (4.18, 4.63)
4.14-5.80 (male)	5.23 (4.96, 5.66)	5.48 (5.33, 5.82)	5.29 (5.01, 5.51)	4.88 (4.78, 5.06)	5.40 (4.99, 5.42)
**WBC, x10^3/µL**					
3.4-10.8	5.4 (4.6, 6.3)	4.7 (4.2, 5.8)	4.5 (3.6, 5.4)	4.8 (4.4, 5.8)	5.4 (4.6, 6.9)
**Neutrophils, %**	56 (50, 61)	54 (48, 59)	48 (43, 56)	54 (50, 60)	57 (54, 65)
**Neutrophils, x10^3/µL**					
1.4-7.0	2.9 (2.5, 3.6)	2.6 (2.0, 3.1)	2.1 (1.5, 3.0)	2.8 (2.3, 3.2)	3.0 (2.6, 4.2)
**Lymphocytes, %**	31 (27, 38)	34 (28, 42)	38 (29, 45)	33 (28, 38)	32 (24, 34)
**Lymphocytes, x10^3/µL**					
0.7-3.1	1.7 (1.2, 2.1)	1.6 (1.3, 1.9)	1.4 (1.3, 1.9)	1.5 (1.3, 2.1)	1.5 (1.3, 2.2)
**Monocytes, %**	8 (7, 10)	10 (8, 11)	10 (9, 12)	8 (7, 10)	8 (7, 9)
**Monocytes, x10^3/µL**					
0.1-0.9	0.5 (0.4, 0.5)	0.5 (0.4, 0.5)	0.4 (0.4, 0.5)	0.4 (0.3, 0.5)	0.4 (0.4, 0.5)
**Eosinophils, %**	2 (2, 3)	2 (1, 3)	3 (2, 3)	2 (1, 3)	2 (1, 3)
**Eosinophils, x10^3/µL**					
0.0-0.4	0.1 (0.1, 0.2)	0.1 (0.1, 0.1)	0.1 (0.1, 0.2)	0.1 (0.1, 0.2)	0.2 (0.1, 0.2)
**Basophils, %**	1 (1, 1)	1 (1, 1)	1 (1, 1)	1 (1, 1)	1 (1, 1)
**Basophils, x10^3/µL**					
0.0-0.2	0.0 (0.0, 0.1)	0.0 (0.0, 0.1)	0.00 (0.0, 0.0)	0.0 (0.0, 0.0)	0.0 (0.0, 0.1)
**Immature Grans, %**	0 (0, 0)	0 (0, 0)	0 (0, 0)	0 (0, 0)	0 (0, 0)
**Immature Grans, x10^3/µL**					
0.0-0.1	0 (0, 0)	0 (0, 0)	0 (0, 0)	0 (0, 0)	0.0 (0.0, 0.0)

Normal reference ranges are listed below the respective variable [3]. At the BL, EOF, EOR visits, there were 29 participants and at the 6wkFU and 12mFU visits there were 26 and 17 participants, respectively. Two participants began refeeding before the EOF blood draw and were excluded from EOF analysis. Due to laboratory errors, one value for platelets was missing from the BL, EOR, and 6wkFU analysis and 2 values for immature grans were missing from EOR analysis. CBC, complete blood count; IQR, interquartile range; g/dL, grams per deciliter; MCV, Mean corpuscular volume; fL, femtoliter; MCH, mean corpuscular hemoglobin; pg, picogram; MCHC, Mean corpuscular hemoglobin concentration; RDW, red cell distribution width; RBC, red blood cell; µL, microliter; WBC, white blood count; Grans, granulocytes; BL, baseline; EOF, end-of-fast; EOR; end-of-refeed; 6wkFU, six-week follow-up; 12mFU, 12-month follow-up.

Table S6. Significance of Differences for CBC

	Estimates (95% CI)						
	**EOF - BL**	**EOR - BL**	**6wkFU - BL**	**12mFU - BL**	**12mFU – 6wkFU**	**EOR – EOF**	**6wkFU – EOR**
[ξ]**Hematocrit, %**	2.62* (1.80, 3.44)	0.35 (-0.46, 1.17)	-0.32 (-1.17, 0.53)	0.79 (-0.19, 1.77)	1.11* (0.11, 2.10)	-2.26* (-3.08, -1.44)	-0.67 (-1.52, 0.18)
**Hemoglobin, g/dL**	1.26* (0.95, 1.57)	0.29 (-0.03, 0.60)	-0.30 (-0.63, 0.02)	0.06 (-0.31, 0.44)	0.36 (-0.02, 0.74)	-0.97* (-1.29, -0.66)	-0.59* (-0.91, -0.26)
**MCV, fL**	-1.24* (-2.02, -0.47)	-0.41 (-1.19, 0.36)	2.57* (1.76, 3.37)	2.70* (1.77, 3.63)	0.13 (-0.81, 1.08)	0.83* (0.05, 1.60)	2.98* (2.18, 3.79)
**MCH, pg**	0.27* (0.01, 0.54)	0.20 (-0.07, 0.46)	0.39* (0.12, 0.66)	0.49* (0.17, 0.81)	0.10 (-0.22, 0.42)	-0.08 (-0.34, 0.19)	0.19 (-0.08, 0.46)
**MCHC, g/dL**	0.75* (0.46, 1.04)	0.33* (0.04, 0.62)	-0.54* (-0.84, -0.24)	-0.46* (-0.81, -0.11)	0.08 (-0.28, 0.43)	-0.41* (-0.70, -0.12)	-0.87* (-1.17, -0.57)
[ξ]**RDW, %**	0.27* (0.07, 0.46)	0.26* (0.07, 0.46)	0.49* (0.29, 0.70)	0.07 (-0.16, 0.31)	-0.42* (-0.66, -0.18)	-0.01 (-0.20, 0.19)	0.23* (0.03, 0.43)

**Platelets, x103/µL**	5.45 (-8.01, 18.78)	-18.46* (-31.88, -5.05)	-12.61 (-26.55, 1.32)	-9.19 (-25.02, 6.78)	3.42 (-12.64, 19.63)	-23.92* (-37.25, -10.46)	5.86 (-8.08, 19.78)
**RBC count, x10^6/µL**	0.38* (0.27, 0.49)	0.07 (-0.04, 0.18)	-0.16* (-0.28, -0.05)	-0.06 (-0.19, 0.07)	0.10 (-0.03, 0.24)	-0.31* (-0.42, -0.20)	-0.23* (-0.34, -0.11)
**WBC, x103/µL**	-0.28 (-0.65, 0.08)	-0.79* (-1.16, -0.43)	-0.32 (-0.69, 0.06)	-0.00 (-0.43, 0.44)	0.32 (-0.12, 0.76)	-0.51* (-0.88, -0.15)	0.48* (0.10, 0.86)
**Neutrophils, %**	-1.72 (-4.28, 0.83)	-5.97* (-8.52, -3.41)	-0.73 (-3.37, 1.94)	2.22 (-0.83, 5.30)	2.95 (-0.16, 6.06)	-4.24* (-6.80, -1.69)	5.24* (2.60, 7.90)
**[ξ] Neutrophils, x10^3/µL**	-0.27* (-0.53, -0.01)	-0.76* (-1.02, -0.50)	-0.23 (-0.50, 0.04)	0.02 (-0.29, 0.34)	0.25 (-0.07, 0.57)	-0.49* (-0.75, -0.23)	0.53* (0.26, 0.80)
**Lymphocytes, %**	1.45 (-0.73, 3.63)	4.31* (2.13, 6.49)	0.39 (-1.88, 2.64)	-1.77 (-4.40, 0.84)	-2.16 (-4.82, 0.49)	2.86* (0.68, 5.04)	-3.92* (-6.19, -1.67)
**Lymphocytes, x103/µL**	0.00 (-0.13, 0.13)	-0.08 (-0.21, 0.05)	-0.08 (-0.21, 0.06)	-0.15 (-0.30, 0.01)	-0.07 (-0.23, 0.09)	-0.08 (-0.21, 0.05)	0.00 (-0.13, 0.14)
**[λ]Monocytes, %**	1.08 (0.91, 1.27)	1.16 (0.98, 1.36)	1.00 (0.84, 1.20)	0.92 (0.75, 1.13)	0.92 (0.74, 1.13)	1.07 (0.91, 1.26)	0.87 (0.73, 1.03)
**[λ]Monocytes[τ], x103/µL**	1.05 (0.83, 1.34)	1.02 (0.81, 1.30)	0.97 (0.75, 1.24)	0.97 (0.73, 1.29)	1.00 (0.75, 1.34)	0.97 (0.77, 1.23)	0.95 (0.74, 1.21)
**[λ]Eosinophils, %**	0.89 (0.63, 1.24)	1.13 (0.82, 1.55)	1.07 (0.76, 1.50)	1.09 (0.74, 1.60)	1.02 (0.69, 1.50)	1.27 (0.92, 1.76)	0.95 (0.68, 1.32)
**[λ]Eosinophils[τ], x103/µL**	0.81 (0.50, 1.31)	0.97 (0.62, 1.54)	1.07 (0.67, 1.69)	1.23 (0.74, 2.05)	1.15 (0.69, 1.92)	1.20 (0.74, 1.94)	1.10 (0.69, 1.75)
**[λ]Basophils, %**	0.93 (0.54, 1.60)	0.96 (0.56, 1.65)	0.95 (0.55, 1.66)	0.94 (0.50, 1.77)	0.99 (0.52, 1.90)	1.04 (0.60, 1.80)	0.99 (0.56, 1.73)
**[‡]Basophils, x10^3/µL**	1.92 (0.38, 9.67)	0.69 (0.13, 3.72)	0.58 (0.10, 3.41)	6.12 (0.79, 47.8)	10.50[α] (1.15, 96.90)	0.34 (0.06, 1.85)	0.79 (0.13, 4.87)
**Immature Grans, %**	NR	NR	NR	NR	NR	NR	NR
**Immature Grans, x103/µL**	NR	NR	NR	NR	NR	NR	NR

CBC, complete blood count; CI, confidence interval; BL, baseline; EOF, end-of-fast; EOR, end-of-refeed; 6wkFU, six-week follow-up; 12mFU, 12-month follow-up; g/dL, grams per deciliter; MCV, Mean Corpuscular Volume; fL, femtoliter; MCH, mean corpuscular hemoglobin; pg, picogram; MCHC, Mean corpuscular hemoglobin concentration; RDW, red cell distribution width; RBC, red blood cell; µL, microliter; WBC, white blood count; Grans, granulocytes; NR, Results not reported due to poor diagnostics. *Zero lies outside the 95% CI so the finding is considered significant. [α]One lies outside the 95% CI so the finding is considered statistically significant. [‡]Logistic regression (included baseline medication-controlled SBP/DBP indicator as a control variable); [λ]Poisson regression; [τ]Measure was multiplied by 10 prior to statistical modeling; [ξ]Used robust mixed-effects model on complete cases.

Table S7. CMP by Visit

	Median (IQR)				
	**BL**	**EOF**	**EOR**	**FU**	**12mFU**
**ALT, IU/L**					
All	19 (15, 26)	22 (17, 32)	26 (20, 36)	18 (14, 26)	18 (15, 21)
0-32 (female)	17 (14, 20)	24 (17, 30)	26 (19, 37)	17 (14, 24)	18 (14, 21)
0-44 (male)	26 (20, 37)	22 (18, 31)	24 (20, 28)	21 (18, 26)	18 (18, 19)
**AST, IU/L**					
0-40	22 (18, 27)	30 (25, 36)	32 (24, 38)	21 (18, 23)	22 (18, 23)
**Albumin, g/L**					
All	46 (44, 48)	47 (45, 49)	45 (43, 47)	46 (42, 46)	44 (42, 47)
38-48 (31-50 y, female)	42 (42, 42)	44 (44, 44)	42 (42, 42)	45 (45, 45)	44 (N/A)
40-50 (31-50 y, male)	50 (50, 50)	50 (50, 50)	47 (47, 47)	N/A	N/A
38-49 (51-60 y)	47 (46, 49)	48 (45, 50)	48 (44, 49)	44 (41, 46)	44 (43, 47)
38-48 (61-70 y)	45 (44, 47)	47 (45, 48)	44 (43, 46)	46 (44, 46)	45 (42, 47)
37-47 (71-80 y)	N/A	N/A	N/A	N/A	43 (42, 44)
**Globulin, g/dL**					
1.5-4.5	2.5 (2.2, 2.7)	2.5 (2.3, 2.9)	2.4 (2.2, 2.7)	2.3 (2.1, 2.6)	2.5 (2.3, 2.8)
**A/G Ratio**					
1.2-2.2	1.9 (1.7, 2.1)	1.9 (1.7, 2.1)	1.9 (1.7, 2.2)	1.9 (1.8, 2.1)	1.9 (1.5, 2.0)
**Alkaline Phosphate, IU/L**					
44-121	87 (70, 96)	90 (70, 104)	82 (67, 94)	91 (84, 101)	91 (71, 106)
**Bilirubin, µmol/L**					
0.0-20.5	10.3 (6.8, 13.7)	15.4 (10.3, 21.4)	6.8 (5.1, 8.6)	8.6 (6.8, 10.3)	6.8 (5.1, 8.6)
**BUN, mg/dL**					
All	9 (8, 10)	8 (7, 10)	6 (5, 8)	8 (7, 10)	14 (13, 17)
6-24 (40-59 y)	9 (8, 10)	8 (7, 10)	7 (6, 7)	10 (8, 10)	13 (10, 16)

8-27 (60-89 y)	9 (8, 10)	8 (7, 10)	6 (4, 8)	8 (7, 10)	10 (9, 13)
**Creatinine, mg/dL**					
All	0.80 (0.77, 0.92)	1.00 (0.84, 1.14)	0.92 (0.77, 1.06)	0.77 (0.67, 0.90)	0.78 (0.64, 0.90)
0.57-1.00 (female)	0.80 (0.72, 0.82)	0.91 (0.77, 1.00)	0.84 (0.75, 0.99)	0.72 (0.65, 0.79)	0.75 (0.63, 0.81)
0.76-1.27 (male)	0.99 (0.81, 1.10)	1.14 (1.04, 1.19)	1.07 (0.94, 1.30)	0.91 (0.84, 1.04)	0.95 (0.88, 0.98)
**BUN/Creatinine Ratio**					
All	11 (9, 13)	8 (7, 11)	7 (6, 9)	11 (9, 13)	14 (13, 17)
9-23 (18-59 y, female)	10 (8, 15)	8 (7, 8)	8 (7, 10)	12 (9, 15)	16 (13, 20)
12-28 (>59 y, female)	11 (9, 13)	8 (7, 11)	6 (5, 7)	12 (9, 13)	16 (13, 17)
9-20 (18-59 y, male)	10 (9, 11)	8 (8, 9)	4 (4, 6)	9 (8, 10)	N/A
10-24 (>59 y, male)	12 (11, 14)	10 (9, 12)	8 (8, 10)	11 (10, 11)	13 (10, 14)
**Calcium, mmol/L**					
All	2.40 (2.33, 2.45)	2.50 (2.42, 2.55)	2.45 (2.40, 2.50)	2.39 (2.29, 2.49)	2.38 (2.34, 2.43)
2.18-2.55 (18-59 y)	2.39 (2.34, 2.52)	2.48 (2.42, 2.55)	2.46 (2.41, 2.49)	2.39 (2.32, 2.41)	2.35 (2.29, 2.41)
2.18-2.56 (>59 y, female)	2.42 (2.35, 2.45)	2.54 (2.45, 2.58)	2.48 (2.40, 2.50)	2.42 (2.37, 2.49)	2.38 (2.36, 2.41)
2.15-2.55 (>59 y, male)	2.35 (2.31, 2.39)	2.48 (2.44, 2.51)	2.42 (2.38, 2.45)	2.34 (2.31, 2.37)	2.38 (2.35, 2.50)
**Carbon Dioxide, mmol/L**					
20-29	24 (21, 26)	17 (15, 20)	25 (23, 26)	24 (22, 26)	24 (23, 24)
**eGFR, mL/min/1.73**					
>59	82 (75, 93)	71 (63, 78)	75 (62, 86)	90 (79, 95)	89 (85, 98)
**Glucose, mg/dL**					
65-99	95 (88, 101)	74 (69, 80)	96 (91, 105)	92 (87, 98)	91 (84, 96)
**Protein, g/dL**					
6.0-8.5	7.0 (6.7, 7.3)	7.3 (7.0, 7.6)	6.9 (6.6, 7.2)	6.7 (6.6, 7.0)	7.0 (6.7, 7.2)
**Potassium, mmol/L**					
3.5-5.2	4.2 (4.1, 4.4)	3.6 (3.4, 3.9)	4.2 (4.1, 4.5)	4.2 (4.1, 4.5)	4.5 (4.0, 4.7)
**Sodium, mmol/L**					
134-144	141 (139, 143)	138 (136, 140)	137 (137, 140)	142 (141, 143)	140 (139, 142)
**Chloride, mmol/L**					
96-106	102 (101, 104)	96 (94, 98)	98 (96, 100)	104 (103, 106)	103 (102, 104)

Normal reference ranges are listed below the respective variable [3]. At the BL, EOF, EOR visits, there were 29 participants and at the 6wkFU and 12mFU visits, there were 26 and 17 participants, respectively. Two participants began refeeding before the EOF blood draw and were excluded from EOF analysis. Due to laboratory errors, 1 value was missing for sodium, potassium, chloride, carbon dioxide, calcium, and ALP at the 12mFU visit. CMP, comprehensive metabolic panel; IQR, interquartile range; BL, baseline; EOF, end-of-fast; EOR, end-of-refeed; 6wkFU, six-week follow-up; 12mFU, 12-month follow-up; ALT, alanine aminotransferase; IU/L, international unit per liter; g/L, gram per liter; g/dL, gram per deciliter; N/A, not applicable; y, years; A/G, Albumin/Globulin; AST, aspartate aminotransferase; µmol/L, micromole per liter; BUN, Blood urea nitrogen; mg/dL, milligram per deciliter; mmol/L, millimol per liter; eGFR, estimated glomerular filtration rate; mL/min, milliliter per minute.

Table S8. Significance of Differences for CMP

	Estimates (95% CI)						
	**EOF -BL**	**EOR - BL**	**6wkFU - BL**	**12mFU - BL**	**12mFU – 6wkFU**	**EOR - EOF**	**6wkFU - EOR**
**ALT, IU/L[‡]**	5.01[*] (1.06, 8.96)	5.68[*] (1.74, 9.63)	-1.36 (-5.44, 2.73)	-2.17 (-6.88, 2.54)	-0.82 (-5.60, 3.96)	0.67 (-3.28, 4.62)	-7.04[*] (-11.13, -2.95)
**AST, IU/L[‡]**	8.34[*] (5.41, 11.27)	7.36[*] (4.43, 10.29)	-1.33 (-4.37, 1.70)	-1.36 (-4.94, 2.22)	-0.02 (-3.65, 3.60)	-0.98 (-3.91, 1.95)	-8.69[*] (-11.73, -5.66)
**Albumin, g/L**	1.24[*] (0.11, 2.38)	-0.66 (-1.79, 0.48)	-1.14 (-2.32, 0.03)	-0.76 (-2.13, 0.58)	0.38 (-1.01, 1.74)	-1.90[*] (-3.03, -0.76)	-0.48 (-1.66, 0.69)
**Globulin, g/dL**	0.11[*] (0.01, 0.21)	-0.04 (-0.14, 0.05)	-0.10 (-0.20, 0.01)	-0.03 (-0.15, 0.09)	0.06 (-0.06, 0.19)	-0.16[*] (-0.25, -0.06)	-0.05 (-0.15, 0.05)
**A/G Ratio**	-0.04 (-0.13, 0.06)	-0.00 (-0.10, 0.09)	0.01 (-0.09, 0.11)	0.00 (-0.11, 0.12)	-0.00 (-0.12, 0.11)	0.03 (-0.06, 0.13)	0.01 (-0.09, 0.11)
**Alkaline Phosphate, IU/L**	3.24 (-0.80, 7.28)	-4.45[*] (-8.49, -0.41)	3.15 (-1.03, 7.35)	4.27 (-0.69, 9.23)	1.12 (-3.91, 6.14)	-7.69[*] (-11.73, -3.65)	7.60[*] (3.42, 11.79)
**Bilirubin, µmol/L[‡]**	4.84[*] (3.59, 6.09)	-2.95[*] (-4.21, -1.70)	-1.54[*] (-2.83, -0.24)	-2.34[*] (-3.84, -0.84)	-0.80 (-2.32, 0.72)	-7.79[*] (-9.04, -6.54)	1.42[*] (0.12, 2.71)
**BUN, mg/dL[‡]**	-0.36 (-1.37, 0.65)	-2.48[*] (-3.49, -1.47)	-0.26 (-1.30, 0.79)	2.00[*] (0.80, 3.20)	2.26[*] (1.04, 3.48)	-2.12[*] (-3.13, -1.11)	2.22[*] (1.18, 3.27)
**Creatinine, mg/dL**	0.15[*] (0.10, 0.20)	0.10[*] (0.05, 0.15)	-0.02 (-0.07, 0.03)	-0.03 (-0.09, 0.02)	-0.01 (-0.07, 0.05)	-0.04 (-0.09, 0.00)	-0.13[*] (-0.18, -0.07)
**BUN/Creatinine Ratio**	-2.17[*]	-4.07[*]	0.34	3.34[*]	3.01[*]	-1.90[*]	4.41[*]

	(-3.66, -0.69)	(-5.55, -2.59)	(-1.19, 1.88)	(1.60, 5.12)	(1.23, 4.80)	(-3.38, -0.41)	(2.88, 5.94)
**Calcium, mmol/L**	0.10[*]	0.06[*]	0.00	-0.04	-0.04	-0.04[*]	-0.06[*]
	(0.06, 0.13)	(0.03, 0.10)	(-0.04, 0.04)	(-0.08, 0.01)	(-0.08, 0.01)	(-0.07, -0.00)	(-0.10, -0.02)
**Carbon Dioxide, mmol/L[‡]**	-5.86[*]	0.85	0.23	0.14	-0.08	6.72[*]	-0.63
	(-7.38, -4.35)	(-0.66, 2.37)	(-1.33, 1.78)	(-1.65, 1.94)	(-1.92, 1.75)	(5.20, 8.23)	(-2.19, 0.93)
**eGFR, mL/min/1.73**	-12.07[*]	-7.93[*]	3.34	6.19[*]	2.84	4.14[*]	11.27[*]
	(-15.96, -8.18)	(-11.82, -4.04)	(-0.69, 7.37)	(1.53, 10.84)	(-1.88, 7.57)	(0.25, 8.03)	(7.24, 15.30)
**Glucose, mg/dL**	-17.10[*]	3.52	-1.38	-4.07	-2.69	20.62[*]	-4.90[*]
	(-21.63, -12.57)	(-1.01, 8.05)	(-6.07, 3.30)	(-9.44, 1.36)	(-8.14, 2.82)	(16.09, 25.15)	(-9.59, -0.21)
**Protein, g/dL**	0.23[*]	-0.11	-0.21[*]	-0.10	0.11	-0.34[*]	-0.10
	(0.09, 0.38)	(-0.26, 0.04)	(-0.36, -0.06)	(-0.27, 0.08)	(-0.07, 0.29)	(-0.49, -0.20)	(-0.25, 0.05)
**Potassium, mmol/L**	-0.55[*]	0.03	0.09	0.15	0.06	0.58[*]	0.06
	(-0.70, -0.39)	(-0.12, 0.18)	(-0.07, 0.25)	(-0.04, 0.34)	(-0.13, 0.25)	(0.43, 0.73)	(-0.10, 0.22)
**Sodium, mmol/L**	-2.79[*]	-2.79[*]	0.74	-0.52	-1.26	0.00	3.53[*]
	(-4.06, -1.53)	(-4.06, -1.53)	(-0.57, 2.04)	(-2.05, 1.00)	(-2.82, 0.29)	(-1.26, 1.26)	(2.23, 4.83)
**Chloride, mmol/L**	-6.10[*]	-4.07[*]	1.52[*]	0.98	-0.54	2.03[*]	5.59[*]
	(-7.39, -4.82)	(-5.35, -2.79)	(0.20, 2.85)	(-0.59, 2.51)	(-2.14, 1.01)	(0.75, 3.32)	(4.27, 6.92)

CMP, comprehensive metabolic panel; CI, confidence interval; BL, baseline; EOF, end-of-fast; EOR, end-of-refeed; 6wkFU, SIX-week follow-up; 12mFU, 12-month follow-up; ALT, alanine aminotransferase; IU/L, international unit per liter; g/L, gram per liter; g/dL, gram per deciliter; N/A, not applicable; y, years; A/G, Albumin/Globulin; AST, aspartate aminotransferase; µmol/L, micromole per liter; BUN, Blood urea nitrogen; mg/dL, milligram per deciliter; mmol/L, millimol per liter; eGFR, estimated glomerular filtration rate; mL/min, milliliter per minute. [*]Zero lies outside the 95% CI so the finding is considered significant. [‡]Used robust mixed-effects model on complete cases.

Table S9. 24-hour Dipstick Urinalysis By Visit

		N (%)		
		**BL**	**EOF**	**EOR**
Leukocytes, cells/µL <15	**0**	26 (90%)	24 (83%)	25 (86%)
	**15**	0 (0%)	1 (3%)	0 (0%)
	**70**	1 (3%)	4 (14%)	2 (7%)
	**125**	2 (7%)	0 (0%)	2 (7%)
Nitrite	**Negative**	24 (83%)	29 (100%)	29 (100%)
	**Positive**	5 (17%)	0 (0%)	0 (0%)
Urobilinogen, µmol/L <17	**0**	19 (66%)	19 (66%)	20 (69%)
	**3.2**	9 (31%)	10 (34%)	9 (31%)
	**16**	1 (3.4%)	0 (0%)	0 (0%)
Protein, mg/dL <30	**0**	27 (93%)	17 (59%)	27 (93%)
	**15**	2 (7%)	9 (31%)	2 (7%)
	**30**	0 (0%)	3 (10%)	0 (0%)
pH	**5.0**	6 (21%)	25 (86%)	8 (28%)
	**6.0**	6 (21%)	4 (14%)	5 (17%)
	**6.5**	9 (31%)	0 (0%)	8 (28%)
	**7.0**	8 (28%)	0 (0%)	6 (21%)
	**7.5**	0 (0%)	0 (0%)	2 (7%)
Blood, cells/µL	**Inconclusive**	1 (3%)[*]	0 (0%)	0 (0%)
	**Negative**	25 (86%)	23 (79%)	26 (90%)
	**Positive**	3 (10%)	6 (21%)	3 (10%)
Specific Gravity	**1.005**	6 (21%)	7 (24%)	12 (41%)
	**1.010**	16 (55%)	11 (38%)	14 (48%)
	**1.015**	7 (24%)	7 (24%)	3 (10%)
	**1.020**	0 (0%)	2 (6.9%)	0 (0%)
	**1.025**	0 (0%)	2 (6.9%)	0 (0%)
	**1.030**	0 (0%)	2 (6.9%)	0 (0%)
Ketone, mmol/L <0.5	**0**	25 (86%)	0 (0%)	27 (93%)
	**0.5**	3 (10%)	1 (3.4%)[†]	0 (0%)

	**1.5**	1 (3.4%)	2 (6.9%)	1 (3.4%)
	**4.0**	0 (0%)	3 (10%)	1 (3.4%)
	**8.0**	0 (0%)	14 (48%)	0 (0%)
	**16**	0 (0%)	9 (31%)	0 (0%)
Bilirubin, µmol/L	**0**	29 (100%)	26 (90%)	29 (100%)
<17	**17**	0 (0%)	3 (10%)	0 (0%)
Glucose, mmol/L	**0**	28 (97%)	29 (100%)	28 (97%)
<5	**5**	1 (3%)	0 (0%)	1 (3.4%)

Normal reference ranges are listed below the respective variable [3]. N (%), number (percent) of participants; BL, baseline; EOF, end-of-fast; EOR, end-of-refeed; µL, microliter; µmol/L, micromole per liter; mg/dL, milligram per deciliter; pH, potential hydrogen; mmol/L, millimol per liter. [*]Participant was menstruating. [†]Participant interrupted majority of the fast with juice.

Table S10. 24-hour Standard Urinalysis by Event

	Median (IQR)			
	**Baseline**	**EOF**	**EOR**	**6wFU**
**Volume, mL**	3,000 (2,750, 3,750)	2,125 (1,494, 2,725)	2,400 (1,900, 2,850)	2,675 (1,962, 3,138)
**Sodium, mmol/L**	20 (20, 26)	20 (20, 20)	20 (20, 20)	20 (20, 25)
**Sodium, mmol/24hr**				
All	70 (58, 85)	42 (30, 54)	49 (38, 58)	60 (45, 82)
39-258 (female)	64 (56, 83)	38 (28, 54)	51 (37, 58)	56 (50, 82)
58-337 (male)	80 (68, 86)	46 (42, 54)	48 (42, 59)	65 (43, 78)
**Protein, mmol/L**	4.20 (4.00, 7.30)	5.8 (4.2, 9.1)	4.60 (4.00, 7.20)	5.6 (4.2, 8.3)
**Protein, mmol/24hr**				
30-150	140 (120, 204)	127 (106, 170)	125 (96, 194)	142 (106, 218)
**Potassium, mmol/L**	31.4 (25.9, 37.9)	11.9 (10.3, 16.0)	29.3 (15.6, 40.0)	32.6 (26.5, 44.0)
**Potassium, mmol/24hr**				
All	100 (74, 130)	26 (21, 32)	62 (37, 84)	93 (67, 110)
14-95 (female)	96 (70, 108)	26 (19, 31)	62 (38, 82)	86 (64, 118)
20-116 (male)	121 (100, 138)	26 (23, 32)	61 (35, 87)	93 (69, 100)
**Albumin, µg/mL**	3.0 (3.0, 3.0)	4.0 (3.0, 6.5)	3.0 (3.0, 3.8)	3.0 (3.0, 3.0)
**Albumin, mg/24hr**				
0-29	10 (8, 12)	9 (8, 11)	8 (7, 11)	8 (6, 11)
**USG**	1.005	1.008	1.0045	
1.005-1.030	(1.005, 1.0065)	(1.006, 1.010)	(1.0040, 1.0055)	n/a

IQR, interquartile range; BL, baseline; EOF, end of fast; EOR, end of refeed; FU, follow up; hr, hour; mL, milliliter; mmol/L, millimol per liter; µg/mL, microgram per liter; mg, milligram; USG, urine specific gravity. Reference ranges for normal values according to LabCorp are given below the respective parameter.

Table S11. Daily Rate of Change for Body Weight, Blood Pressure, and Vital Signs

	Rate of Change/Day (N_{obs}, SE, P-value)		
	**Prefeed**	**Fasting**	**Refeed**
**BW, kg**	-1.11 (47, 0.18, <0.0001)[*§]	-0.54 (342, 0.03, <0.0001)[*]	0.23 (174, 0.03, <0.0001)[*]
Early period[‡]	N/A	-0.89 (342, 0.03, <0.0001)[*]	N/A
Late period[‡]	N/A	-0.43 (342, 0.03, <0.0001)[*]	N/A
**Pulse, min^{-1}**	-1.35 (49, 1.25, 0.3)[§]	0.55 (350, 0.16, 0.001)	-0.14 (184, 0.19, 0.5)[‡]
**SpO_2, %**	0.21 (45, 0.22, 0.4)[†§]	0.01 (338, 0.01, 0.3)[§]	-0.01 (177, 0.02, 0.6)[§]
**BT, °C**	-0.01 (47, 0.05, 0.9)[*§]	-0.01 (349, 0.00, <0.0001)[§]	0.01 (181, 0.00, 0.02)[§]
**SBP, mmHg**	-9.45 (22, 7.45, 0.252)	-1.21 (351, 0.27, <0.0001)	0.32 (184, 0.22, 0.146)[§]

**DBP, mmHg**	-3.50<br>(22, 2.96, 0.282)	-0.29<br>(351, 0.13, 0.026)	0.21<br>(184, 0.12, 0.073) §

N = 27 participants. N_{obs}, Total number of observations in model; SE, Standard error of measurement; P-value, probability value (≤ 0.05 is considered significant); BW, body weight; kg, kilogram; min-1, per minute; SpO2, saturation of peripheral oxygen; %, percent; BT, body temperature; °C, degrees Celsius; N/A, not applicable. *26 participants. †24 participants. ‡Estimated daily rate of change of BW in early period (day 1-5) and late period (>5 days) of fasting. §Model excluded random slope.

Table S12. Weight Class by Visit

Category<br>BMI (kg/m²)	BL	EOF	EOR	6wkFU	12mFU
	N* (%)				
**Underweight**<br>< 18.5	0 (0)	1 (3)	1 (3)	0 (0)	0(0)
**Normal**<br>18.5-24.9	4 (14)	8 (28)	8 (28)	8 (32)	5 (29)
**Overweight**<br>25-29.9	10 (34)	11 (38)	9 (31)	10 (40)	7 (41)
**Obese**<br>> 30	15 (52)	9 (31)	11 (38)	7 (28)	5 (29)
**Obese I**<br>30-34.9	10 (34)	7 (24)	9 (31)	7 (28)	3 (18)
**Obese II**<br>> 34.9	5 (17)	2 (7)	2 (7)	0 (0)	2(12)

BMI (kg/m²) reference ranges for weight categories are listed below respective variable [4]. N (%), number (percent) of participants in respective category; BMI, body mass index; kg/m², kilogram per square meter; BL, baseline; EOF, end-of-fast; EOR, end-of-refeed; 6wkFU, six-week follow-up; 12mFU, 12-month follow-up. *At the BL, EOF, EOR visits, there were 29 participants and at the 6wkFU and 12mFU visits, there were 26 and 17 participants, respectively.

Table S13. Cardiometabolic Markers by Visit

	BL	EOF^	EOR	6wFU#	12mFU
	Median (IQR)				
**BW, kg**	86.6 (80.1, 97.6)	79.1 (72.4, 87.8)	81.4 (73.8, 88.7)	80.3 (75.3, 85.7)	80.4 (72.1, 88.9)
**BMI, kg²/m²**<br>18.5-24.9	31.0 (26.5, 33.2)	28.0 (23.6, 30.1)	28.4 (24.4, 30.8)	29.0 (24.2, 30.2)	28.4 (23.5, 31.1)
**AC, cm**					
**All**	100.5 (92.0, 109.0)	91.5 (86.4, 101.0)	94.3 (88.0, 102.0)	94.9 (87.0, 102.0)	90.6 (85.0, 95.0)
Female: <88<br>Male: <102	100.5 (91.8, 108.7)	91.5 (86.7, 101.0)	93.5 (88.1, 102.0)	94.3 (86.5, 101.3)	90.3 (86.1, 93.7)

	100.3 (95.7, 107.3)	90.8 (85.8, 100.0)	95.0 (87.4, 102.6)	96.1 (91.0, 102.8)	91.0 (81.3, 95.0)
**SBP, mmHg**<br><120	136 (129, 146)	125 (120, 131)	114 (108, 123)	121 (113, 138)	127 (116, 132)
**DBP, mmHg**<br><80	84 (77, 88)	84 (80, 85)	78 (74, 82)	78 (74, 83)	73 (71, 76)
**Total Cholesterol, mmol/L**<br>2.59 - 5.15	5.26 (4.35, 5.80)	5.39 (4.66, 6.54)	4.71 (4.12, 5.52)	4.97 (4.38, 5.44)	4.92 (4.38, 6.16)
**HDL Cholesterol, mmol/L**<br>> 1.01	1.19 (0.98, 1.42)	1.11 (0.97, 1.31)	1.14 (0.96, 1.40)	1.20 (0.96, 1.42)	1.42 (1.06, 1.50)
**VLDL Cholesterol, mmol/L**<br>0.13 - 1.04	0.57 (0.47, 0.75)	0.57 (0.49, 0.66)	0.67 (0.62, 0.88)	0.60 (0.49, 0.77)	0.52 (0.41, 0.62)
**LDL Cholesterol, mmol/L**<br>< 2.56	3.24 (2.64, 3.81)	3.76 (2.82, 4.48)	2.90 (2.25, 3.60)	3.15 (2.61, 3.58)	3.00 (2.43, 3.86)
**Triglycerides, mmol/L**<br>< 1.68	1.41 (1.08, 1.84)	1.42 (1.18, 1.58)	1.71 (1.50, 2.19)	1.46 (1.15, 1.87)	1.21 (0.96, 1.53)
**Insulin, pmol/L**<br>16 -149	42 (34, 78)	25 (17, 38)	52 (40, 79)	59 (31, 75)	51 (32, 63)
**ln (Insulin, pmol/L)**<br>2.7 - 5.0	3.7 (3.5, 4.4)	3.2 (2.8, 3.6)	4.0 (3.7, 4.4)	4.1 (3.4, 4.3)	3.9 (3.5, 4.1)
**HOMA-IR**<br>< 1.9	1.7 (1.3, 2.9)	0.7 (0.5, 1.3)	2.1 (1.4, 3.4)	2.3 (1.1, 3.1)	2.0 (1.0, 2.6)
**ln (HOMA-IR)**<br>< 0.64	0.51 (0.23, 1.06)	-0.33 (-0.64, 0.24)	0.73 (0.34, 1.22)	0.83 (0.13, 1.13)	0.69 (0.03, 0.95)
**GGT, nmol/(s·L)**<br>< 1000	317 (233, 433)	267 (208, 383)	267 (200, 400)	292 (238, 408)	217 (183, 400)

**ln (GGT, nmol/(s·L))** < 6.90	5.76 (5.45, 6.07)	5.59 (5.34, 5.95)	5.59 (5.30, 5.99)	5.68 (5.47, 6.01)	5.38 (5.21, 5.99)
**Fatty Liver Index** < 30	60.1 (38.5, 84.9)	39.2 (19.3, 60.1)	54.6 (26.0, 74.6)	48.1 (23.6, 70.7)	34.9 (23.0, 52.0)
**hsCRP, mg/L** < 3.0	1.67 (0.65, 3.40)	3.09 (1.23, 4.87)	1.32 (0.64, 3.04)	0.84 (0.63, 1.49)	1.03 (0.65, 1.95)
**ln (hsCRP, mg/L)** < 1.09	0.51 (-0.43, 1.22)	1.13 (0.20, 1.58)	0.28 (-0.45, 1.11)	-0.18 (-0.46, 0.40)	0.03 (-0.43, 0.67)

Normal reference ranges are listed below each variable [3]. At BL, EOF, and EOR, there were 29 participants. At the 6wFU and 12mFU, there were 26 and 17 participants, respectively. IQR, interquartile range; BL, baseline; EOF, end-of-fast; EOR, end-of-refeed; 6wFU, six-week follow-up; 12mFU, 12-month follow-up; BW, body weight; BMI, body mass index; kg/m^2, kilogram per square meter; AC, abdominal circumference; cm, centimeter; SBP, systolic blood pressure; DBP, diastolic blood pressure; mmHg, millimeter mercury; kg, kilogram; LDL, low-density lipoprotein; HDL, high-density lipoprotein; VLDL, very-low-density lipoprotein; mmol/L, millimole per liter; pmol/L, picomole per liter; HOMA-IR, homeostatic model assessment for insulin resistance; GGT, gamma-glutamyl transferase; nmol/(s·L), nanomole per second and liter; hsCRP, high-sensitivity C-reactive protein; mg/L, milligram per liter; ln, natural logarithm. ^Two participants began refeeding before EOF blood draw and were excluded from all serology. #Three participants did not excluded from the 6wFU analyses and of the remaining 26, one only provided values for blood pressure. 18 of participants data previously reported in

Table S14. Significance of Differences for Cardiometabolic Markers

	Estimates (95% CI)						
	EOF - BL	EOR - BL	6wFU - BL	12mFU - BL	12mFU – 6wFU	EOR - EOF	6wFU - EOR
**Weight, kg‡**	-7.76* (-8.84, -6.67)	-6.59* (-7.68, -5.50)	-6.72* (-7.87, -5.58)	-5.41* (-6.72, -4.11)	1.31 (-0.02, 2.64)	1.17* (0.08, 2.25)	-0.13 (-1.28, 1.01)
**BMI, kg/m2**	-2.73* (-3.41, -2.05)	-2.33* (-3.01, -1.65)	-2.58* (-3.29, -1.86)	-2.67* (-3.49, -1.86)	-0.09 (-0.93, 0.74)	0.40 (-0.28, 1.08)	-0.25 (-0.97, 0.46)
**AC, cm‡**	-7.45* (-8.83, -6.07)	-5.94* (-7.32, -4.56)	-6.55* (-8.00, -5.10)	-7.92* (-9.58, -6.26)	-1.37 (-3.06, 0.33)	1.51* (0.13, 2.89)	-0.61 (-2.06, 0.84)
**SBP, mmHg**	-10.24* (-15.84, -4.64)	-19.83* (-25.43, -14.23)	-10.24* (-16.04, -4.46)	-8.80* (-15.47, -2.15)	1.43 (-5.33, 8.21)	-9.59* (-15.19, -3.99)	9.59* (3.79, 15.37)
**DBP, mmHg**	-0.21	-5.24*	-4.65*	-8.83*	-4.17*	-5.03*	0.59

	(-3.17, 2.76)	(-8.21, -2.28)	(-7.71, -1.57)	(-12.35, -5.27)	(-7.76, -0.58)	(-8.00, -2.07)	(-2.47, 3.67)
**Total Cholesterol, mol/L**	0.30 (-0.03, 0.63)	-0.34* (-0.67, -0.02)	-0.26 (-0.60, 0.08)	0.10 (-0.30, 0.48)	0.35 (-0.05, 0.75)	-0.64* (-0.97, -0.32)	0.08 (-0.26, 0.42)
**LDL, mmol/L**	0.38* (0.09, 0.67)	-0.42* (-0.71, -0.14)	-0.23 (-0.53, 0.07)	0.04 (-0.31, 0.38)	0.27 (-0.09, 0.62)	-0.80* (-1.09, -0.51)	0.19 (-0.11, 0.49)
**HDL, mmol/L**	-0.06 (-0.15, 0.03)	-0.04 (-0.13, 0.05)	-0.03 (-0.12, 0.07)	0.11* (0.00, 0.22)	0.14* (0.03, 0.24)	0.02 (-0.07, 0.10)	0.02 (-0.08, 0.11)
**VLDL, mmol/L**	-0.02 (-0.10, 0.06)	0.12* (0.04, 0.20)	-0.00 (-0.08, 0.08)	-0.06 (-0.15, 0.03)	-0.05 (-0.15, 0.04)	0.14* (0.06, 0.22)	-0.13* (-0.21, -0.05)
**Triglycerides, mmol/L**	-0.06 (-0.26, 0.13)	0.33* (0.14, 0.53)	0.00 (-0.20, 0.20)	-0.16 (-0.39, 0.07)	-0.16 (-0.40, 0.07)	0.40* (0.20, 0.59)	-0.33* (-0.53, -0.13)
**Insulin, pmol/L‡**	-19.19* (-28.72, -9.65)	11.14* (1.60, 20.68)	7.12 (-2.76, 17.01)	2.98 (-8.44, 14.39)	-4.15 (-15.73, 7.43)	30.33* (20.79, 39.87)	-4.02 (-13.90, 5.87)
**Log Insulin, pmol/L**	-0.54* (-0.81, -0.28)	0.21 (-0.06, 0.47)	0.12 (-0.15, 0.39)	-0.07 (-0.38, 0.25)	-0.19 (-0.51, 0.13)	0.75* (0.49, 1.01)	-0.09 (-0.36, 0.19)
**HOMA-IR‡**	-0.95* (-1.36, -0.54)	0.48* (0.07, 0.89)	0.24 (-0.18, 0.67)	0.07 (-0.42, 0.56)	-0.17 (-0.67, 0.33)	1.43* (1.02, 1.84)	-0.23 (-0.66, 0.19)
**Ln [HOMA-IR]**	-0.75* (-1.04, -0.46)	0.24 (-0.05, 0.53)	0.10 (-0.19, 0.40)	-0.11 (-0.45, 0.23)	-0.21 (-0.56, 0.13)	0.99* (0.70, 1.28)	-0.14 (-0.43, 0.16)
**GGT, nmol/(s*L)‡**	-22.86 (-51.31, 5.58)	-42.34* (-70.79, -13.90)	-7.21 (-36.71, 22.30)	-42.28* (-76.44, -8.12)	-35.07* (-69.70, -0.45)	-19.48 (-47.93, 8.96)	35.14* (5.63, 64.64)
**Ln[GGT, nmol/(s*L)]**	-0.08 (-0.18, 0.02)	-0.11* (-0.21, -0.01)	-0.01 (-0.12, 0.09)	-0.13* (-0.25, -0.01)	-0.12	-0.03	0.10

					(-0.24, 0.01)	(-0.13, 0.07)	(-0.01, 0.20)
**FLI**	-15.54* (-20.62, -10.45)	-9.30* (-14.39, -4.21)	-12.22* (-17.57, -6.87)	-19.28* (-25.41, -13.18)	-7.06* (-13.31, -0.85)	6.23* (1.14, 11.32)	-2.92 (-8.27, 2.43)
**hsCRP, mg/L‡**	1.19* (0.69, 1.69)	-0.31 (-0.81, 0.19)	-0.53* (-1.04, -0.01)	-0.16 (-0.76, 0.43)	0.36 (-0.24, 0.97)	-1.50* (-2.00, -1.00)	-0.22 (-0.73, 0.30)
**Ln[hsCRP, mg/L}**	0.48* (0.13, 0.83)	-0.14 (-0.49, 0.21)	-0.39* (-0.76, -0.03)	0.03 (-0.40, 0.45)	0.42 (-0.01, 0.84)	-0.62* (-0.97, -0.27)	-0.25 (-0.62, 0.11)

CI, confidence interval; BL, baseline; EOF, end-of-fast; EOR, end-of-refeed; 6wFU, six-week follow-up; 12mFU, 12-month follow-up; BW, body weight; BMI, body mass index; kg/m2, kilogram per square meter; AC, abdominal circumference; cm, centimeter; SBP, systolic blood pressure; DBP, diastolic blood pressure; mmHg, millimeter mercury; kg, kilogram; LDL, low-density lipoprotein; HDL, high-density lipoprotein; VLDL, very-low density lipoprotein; mmol/L, millimole per liter; pmol/L, picomole per liter; HOMA-IR, homeostatic model assessment for insulin resistance; GGT, gamma-glutamyl transferase; nmol/(s·L), nanomole per second and liter; FLI; fatty liver index; hsCRP, high-sensitivity C-reactive protein; mg/L, milligram per liter; ln, natural logarithm. *Zero lies outside the 95% CI so the finding is considered significant. ‡Used robust mixed effects model on complete cases.

Table S15. Anti-Hypertensive Medication Use at BL, 6wkFU, and 12mFU

	#		
**Medications**	**BL**	**6wkFU**	**12mFU**
Amlodipine	3	0	2
Amlodipine and Lisinopril	1	0	0
Amlodipine and Spironolactone	0	0	1
Atenolol and Losartan	1	0	0
HCTZ†	2	0	0†
Lisinopril†	1	0	-
Lisinopril/HTCZ*†	2	0*	-
Losartan†	4	1	3†
Propranolol	1	0	0
**Medicated Participants (% of total population)**	**15 (52)**	**1 (4)**	**6 (35)**

At BL, 15/29 participants were taking anti-hypertensive medications, 11/15 were taking a single medication, 2/15 were taking 2 medications (Lisinopril and Amlodipine, Atenolol and Losartan) and 2/15 were taking a combination (Lisinopril/HTCZ). At 6wkFU, 1/26 was taking medication, and at 12mFU, 6/17 participants were taking medications, of which 1/6 was taking 2 medications (Amlodipine and Spironolactone). BL, baseline; 6wkFU, six-week follow-up; 12mFU, 12-month follow-up; HTCZ, Hydrochlorothiazide. *1/15 and †5/15 of the participants taking medications at BL did not participate at 6wkFU and 12mFU, respectively.

2. Supplementary Figures

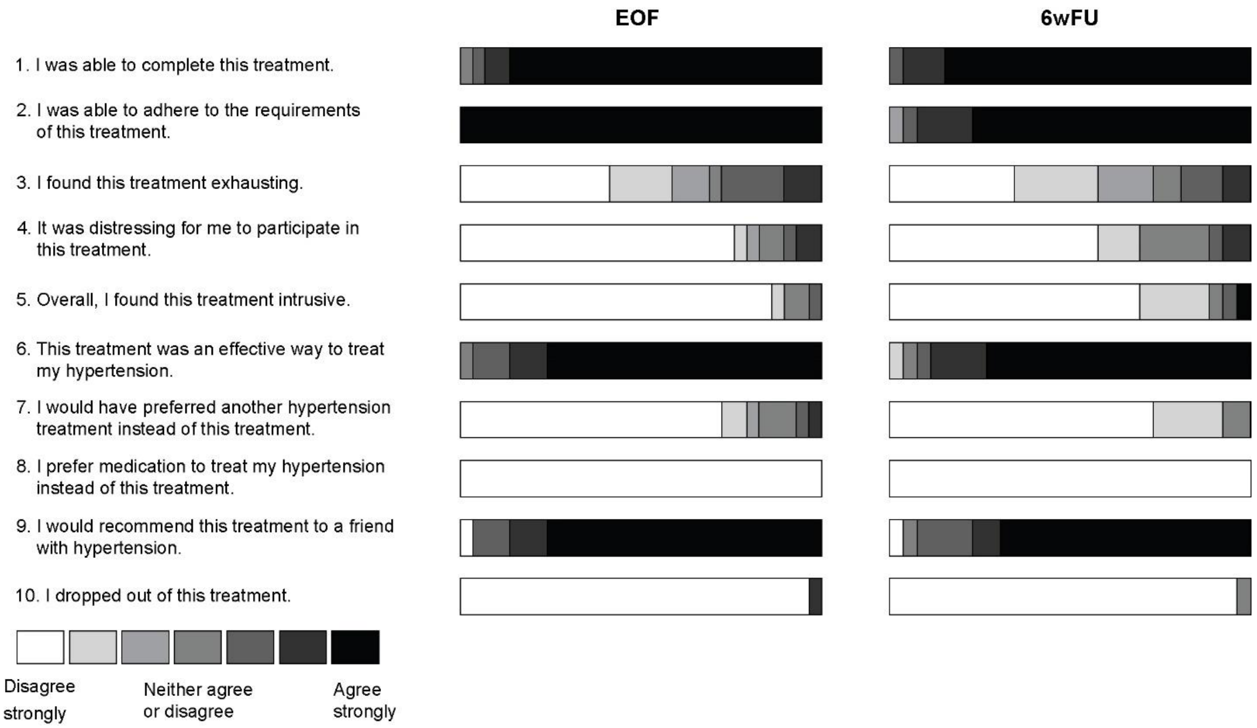

Figure S1: Percentage of participants responding to individual TAAS questions. Presented on scale from white (1, disagree strongly) to medium grey (4, neither agree nor disagree) to black (7, agree strongly). There were 29 and 26 participants at EOF and 6wkFU, respectively. EOF, end-of-fast; 6wkFU, six-week follow-up.

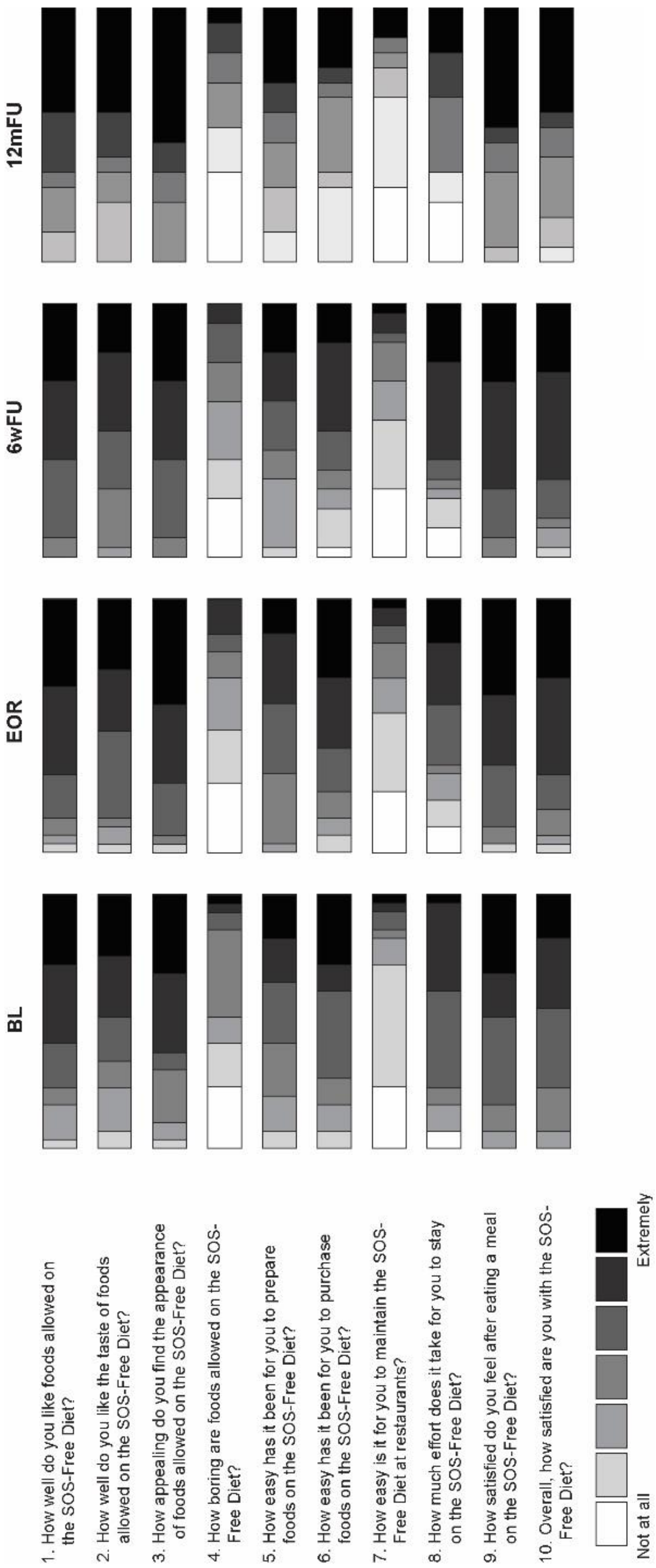

Figure S2: Percentage of participants responding to individual FAQ questions. Presented on scale from white (1, not at all) to black (7, extremely). There were 29, 29, 26, and 17 participants at BL, EOR, 6wkFU, and 12mFU, respectively. BL, baseline; EOR, end-of-refeed; 6wkFU, six-week follow up; 12mFU, 12-month follow up; SOS-Free Diet, exclusively whole-plant-food diet free of added salt, oil and sugar.

References

[1] ICD10Data.
[2] NIH. Common Terminology Criteria for Adverse Events (CTCAE).
[3] LabCorp, Blood Specimens: Chemistry and Hematology.
[4] C.B. Weir, and A. Jan, BMI Classification Percentile And Cut Off Points, StatPearls, Treasure Island (FL), 2023.
